# Mechanisms of Seizure Propagation in 2-Dimensional Centre-Surround Recurrent Networks

**DOI:** 10.1371/journal.pone.0071369

**Published:** 2013-08-13

**Authors:** David Hall, Levin Kuhlmann

**Affiliations:** 1 Victoria Research Labs, National ICT Australia, Parkville, Victoria, Australia; 2 Department of Electrical and Electronic Engineering, The University of Melbourne, Parkville, Victoria, Australia; Indiana University, United States of America

## Abstract

Understanding how seizures spread throughout the brain is an important problem in the treatment of epilepsy, especially for implantable devices that aim to avert focal seizures before they spread to, and overwhelm, the rest of the brain. This paper presents an analysis of the speed of propagation in a computational model of seizure-like activity in a 2-dimensional recurrent network of integrate-and-fire neurons containing both excitatory and inhibitory populations and having a difference of Gaussians connectivity structure, an approximation to that observed in cerebral cortex. In the same computational model network, alternative mechanisms are explored in order to simulate the range of seizure-like activity propagation speeds (0.1–100 mm/s) observed in two animal-slice-based models of epilepsy: (1) low extracellular 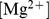, which creates excess excitation and (2) introduction of gamma-aminobutyric acid (GABA) antagonists, which reduce inhibition. Moreover, two alternative connection topologies are considered: excitation broader than inhibition, and inhibition broader than excitation. It was found that the empirically observed range of propagation velocities can be obtained for both connection topologies. For the case of the GABA antagonist model simulation, consistent with other studies, it was found that there is an effective threshold in the degree of inhibition below which waves begin to propagate. For the case of the low extracellular 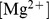 model simulation, it was found that activity-dependent reductions in inhibition provide a potential explanation for the emergence of slowly propagating waves. This was simulated as a depression of inhibitory synapses, but it may also be achieved by other mechanisms. This work provides a localised network understanding of the propagation of seizures in 2-dimensional centre-surround networks that can be tested empirically.

## Introduction

Epilepsy is a debilitating disorder affecting roughly 1–3% of the population [1]. Approximately 33% of these people suffer from pharmaco-resistant epilepsy [2]. Current treatment for pharmaco-resistant epilepsy involves surgical resection of the seizure-generating tissue [3] or, more recently through clinical trials, the implantation of a seizure control device that can avert seizures through electrical stimulation or drug delivery [4]–[6]. The success of both these forms of treatment is heavily dependent on being able to determine the seizure-focus and the epileptic brain network through which seizures first spread before they take over the activity within the rest of the brain. With this in mind, this paper investigates a computational model of the local mechanisms of seizure propagation across a 2-dimensional (2D) centre-surround network of integrate-and-fire (IAF) neurons which can be considered to be a simplified model of the cerebro-cortical sheet.

The neural modelling of seizures has been investigated at several scales (see [7], [8] for reviews). Three key aspects of modelling seizures are describing the mechanisms involved in (1) seizure initiation, (2) seizure propagation, and (3) seizure termination. As mentioned above, this paper is focused on seizure propagation. Specific investigations of the neural modelling of seizure propagation have focused on both the macro-scale [9]–[11] and the scale of a local network-of-neurons [7], [12], [13]. Here we choose to model the local network-of-neurons scale to better understand the local mechanisms of seizure propagation. This knowledge can then be applied to the holy grail problem of better defining macro-scale models of seizure propagation that could be used to determine the paths, and the spatio-temporal sequences, that seizures take through an individual epileptic patient’s brain. Specifically, we have chosen to follow on from the work of Ursino and La Cara [12] who demonstrated different types of travelling wave behaviour during simulated seizures in a 2D centre-surround network of IAF neurons. Our work provides a slightly more realistic network topology including both excitatory and inhibitory neurons and seeks to better understand the mechanisms underlying seizure propagation through comparison with physiological data.

Investigations of seizure spread *in vivo* have demonstrated, typically with intra-cranial electroencephalography (EEG) in humans, that propagation velocities, propagation patterns and connectivity networks can be obtained to a certain degree of accuracy [14]–[18]. However, it is difficult to fully investigate local mechanisms of seizure propagation *in vivo*. *In vitro* slice-studies on the other hand can more easily tease apart local network mechanisms. Two *in vitro* slice models of the propagation of seizure-like activity are the (1) low extracellular 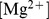 [19]–[23] and (2) gamma-aminobutyric acid (GABA) antagonist [24]–[26] models.

The low extracellular 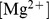 model produces spontaneous seizure-like activity in slices of mouse primary visual cortex with propagation speeds of the order of 0.1–10 mm/s [22], [23], [27]. Low extracellular 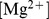 is thought to have its greatest effect on the excitability of NMDA receptors by increasing their probability of remaining open. In cerebral cortex, NMDA receptors lie on both the pre- and post-synaptic regions of an excitatory synapse and therefore low extracellular 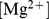 is likely to have an effect on excitability of both parts of the synapse [28], [29]. The seizure-like activity of this animal slice model [22] is reminiscent of the ‘Jacksonian March’ observed in tonic-clonic seizures in humans where seizures progress from one region to another in a step-like manner, as opposed to a smoothly propagating wavefront [30]–[32].

GABA antagonist slice models [24]–[26] predominantly act by preventing GABA from binding to GABA receptors in the synaptic cleft, thus reducing inhibition and allowing excitatory activity to build up into spontaneous seizure-like activity. The lack of inhibition in these models allows for runaway excitation leading to seizure-like activity propagation speeds of up to 100 mm/s [33], [34].

In this paper, through simulations of a computational model of 2D IAF centre-surround networks we provide possible explanations of the mechanisms giving rise to the range of propagation speeds, 0.1 to 100 mm/s, seen in these slice models of epilepsy. In particular, for the case of high propagation speeds seen with GABA antagonist models, as is done in other computational studies [12], [35]–[38], we propose the straight forward mechanism that inhibition is greatly reduced by the blocking of inhibitory (GABA) synapses in the whole network allowing excitation to spread rapidly from a focal region (see discussion for comparison with other computational studies). With regard to the GABA antagonist models, the novelty in this paper lies in a more detailed analysis of the 2D network dynamics.

Alternatively we propose that slow propagation speeds obtained with low extracellular 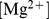 arise as a result of a confluence of factors: (1) pre-synaptic effects on excitatory (NMDA) receptors [28] cause a decrease in the rate of pre-synaptic adaptation of excitatory synapses, (2) post-synaptic effects on excitatory (NMDA) receptors increase post-synaptic excitatory conductance [29], (3) cells in the inhibitory surround initially have enough activity to prevent the spread of excitatory activity but inhibitory synapses adapt faster than excitatory synapses and excitation eventually spreads, and (4) when excitation spreads to a new area the inhibitory surround has to adapt before excitation can spread to the next area, thus producing a ‘Jacksonian March’. Furthermore, for computational simulations of the two animal slice models considered, we explore the influence of connection topologies (excitation broader than inhibition and inhibition broader than excitation) on seizure propagation. We also explore what the minimal necessary conditions on the parameters are for ‘Jacksonian March’-type seizure spread to occur.

The main findings of this paper are: (1) for the GABA antagonist simulations there is an effective threshold of inhibition (or more correctly the balance of excitatory and inhibitory strengths) below which activity begins to spread across the network; (2) for the low extracellular 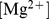 simulations the primary way to produce slowly propagating seizures is to incorporate activity-dependent suppression of inhibition, which has been achieved here through the influence of inhibitory pre-synaptic depression; and (3) by making simple adjustments to the same computational network model for either connection topology we are able to simulate the full range of average seizure propagation velocities observed in the two animal slice preparations considered.

## Methods

The computational model used is based on equations describing the leaky IAF neuron and, as stated in the introduction, is an extension of the work done by Ursino and La Cara [12]. In contrast to Ursino and La Cara, our model includes separate populations of inhibitory and excitatory neurons. We have also included presynaptic depression in our neuronal model.

To begin with, a description of how a single neuron is modelled is provided, followed by an outline of how a network of these neurons is connected and finally an explanation of how the parameters change from ‘normal’ values to describe approximations to the (1) the low extracellular 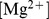 model and (2) the GABA antagonist model.

### Single Neuron

The IAF model reduces the complexity needed to describe the behaviour of a single neuron by replacing the exact dynamic description of the ionic channels involved in the generation of the action potential, with a threshold mechanism [12].

Each neuron contains excitatory and inhibitory synaptic conductances and an after-hyperpolarization conductance in order to describe the refractoriness of the neuron. The mathematical equations that describe the time evolution of the membrane potential of a neuron are as follows:
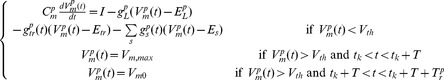
(1)Where 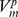, 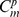 are the membrane voltage and capacitance of the neuron respectively. 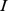 is an external current used to stimulate the neuron. 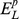 and 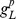 are the leak reversal potential and conductance. 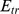 and 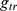 are the after-hyperpolarization reversal potential and conductance. 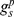 and 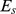 are the synaptic conductances and reversal potentials for the neuron and 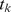 is the instant of the 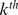 spike. The parameter 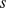 represents whether a synaptic conductance is excitatory (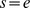) or inhibitory (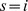) and the parameter 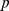 indicates whether the neuron is an excitatory neuron (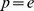) or an inhibitory neuron (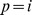).

The differential equation holds until the membrane potential 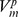 reaches some threshold value 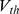 at which point the neuron spikes for a period of 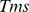 with the membrane potential having a constant value of 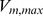. After the spiking period has ended the membrane potential is set to the reset potential 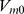 for a period of 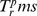 to model the refractory period of the neuron which is the time the neuron needs to rest before it can fire again. This is a step-wise-continuous approximation to the shape of an action potential.

The presence of the after-hyperpolarization conductance, 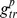 simulates the relative refractoriness period and the phenomenon of spike rate adaptation. It behaves according to the following dynamics:
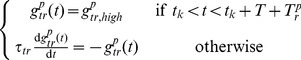
(2)


The excitatory and inhibitory synaptic conductances, 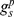, model the voltage gated ion channels in the cell membrane. These channels can be described using a Hodgkin-Huxley-type model where:
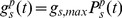
(3)Where 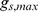 is the conductance when all ionic channels are open and 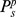 is the probability that those channels are open. This probability can be expressed as a product of two terms 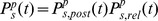. The factor 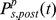 is the probability a postsynaptic channel opens given transmitter was released from a presynaptic terminal and 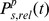 is the probability that transmitter was released from a presynaptic terminal following an action potential [39].

By incorporating 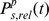 into the dynamics of the system it is possible for us to model presynaptic depression. This occurs when the release probability is reduced after a presynaptic action potential 
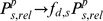
 where the parameter 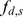 with 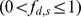 controls the amount of depression. Between presynaptic action potentials 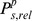 is governed by the following equation:
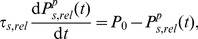
(4)where 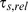 controls the rate at which the release probability is restored to the resting probability 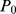. Presynaptic depression was included into the model in order to simulate low extracellular 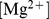 slices (Note it is also simulated in the GABA antagonist simulations). Epileptic networks in general are also likely to show presynaptic facilitation. Facilitation was not included in order to better understand the influences of presynaptic depression alone.

Since there are a large number of postsynaptic channels, 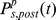 is also a measure of the proportion of channels that are actually open. The rate at which the proportion of open channels changes is determined by the following differential equation:
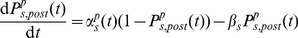
(5)where 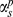 is the rate at which the channels open and 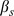 is the rate at which the channels close.

To account for excitatory and inhibitory input from other neurons we assume our opening rate 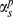 depends on the synaptic input from other neurons. If we require our opening rate to be zero for no synaptic input and for it to increase to some saturation level as synaptic input increases then the opening rate is influenced by the synaptic input as follows:
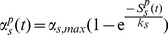
(6)where 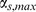 is the maximum opening rate, 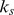 determines the rate of saturation of the synaptic conductance and 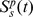 is the synaptic input from the other neurons and is defined below.

### Network Connectivity

The network consists of a 50×50 array of excitatory neurons and a 25×25 array of inhibitory neurons since the observed ratio of excitatory to inhibitory neurons is 4∶1 [40]. Both neuron populations occupy the same spatial extent of 2 mm×2 mm, approximating a small patch of cortex. The physical dimensions are approximately a 16∶1 scaling of the physiological data of Trevelyan et al. [22] where calcium imaging videos showed 152 active neurons occupying a 0.52 mm×0.52 mm area of mouse cortex. These cell densities are an underestimate because there are likely to be tens to hundreds of thousands of neurons across the cortical layers within a 2 mm by 2 mm patch of mouse cortex. Even for just a single cortical layer within cortex these cell densities will likely be an underestimate, however, due to computational constraints it is difficult to simulate accurate cell densities in reasonable amounts of time. Regardless, the important aspect here is appropriate synaptic weighting for the cell densities we apply as well as the appropriate spacing of the neurons and setting of the standard deviations of the Gaussian connectivity kernels (see below) in order to obtain reasonably accurate estimates of velocity. The dimensions we use are also supported by the computational models of Somers et al. and Troyer et al. [41], [42].

Neurons are arranged in a regular lattice with their location in the lattice being represented by the indices 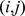. The network was connected with a centre-surround topology. It is well known that surround inhibition exists within cortex on a functional level [36], [43], however, it is not completely clear how this functional property is obtained through anatomical connectivity. In particular, it is known that long range connections can only be excitatory and inhibitory cells can only have short range connections. This appears true in both the hippocampus [44]–[47] and visual cortex [48]–[51]. This leads one to ask, how can an inhibitory surround exist if excitation appears to be anatomically broader than inhibition? Given this uncertainty two connectivity structures were considered, the first being a classical Mexican-Hat topology which is consistent with certain models of cortex [12], [52], [53]. This means a neuron receives excitatory and inhibitory inputs from nearby neurons, with excitation having narrower extension than inhibition. The second connectivity structure considered is the case where excitation is broader than inhibition as outlined by Traub and Miles [36].

It was assumed that the synaptic connections between neurons in the network decreased with distance, 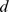. Furthermore, in order to account for the intrinsic variability of synapses, it was assumed that the synaptic strength is weighted by a random factor drawn from a uniform distribution. This signifies that not all neurons receive the same excitatory and inhibitory connection weights. The neuron at position 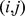 receives synaptic input 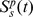 as follows:
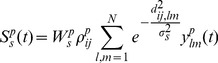
(7)

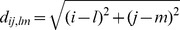
(8)Where 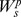 is the synaptic strength, 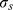 is the standard deviation and represents the spatial extent to which inputs can act, 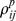 is a uniformly distributed random variable between 0.5 and 1.5 (values were kept fixed during simulations) and 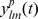 is a quantity that indicates whether the pre-synaptic neuron at position 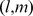 is spiking at time 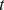 since it is only the spiking neurons that contribute to the synaptic input.
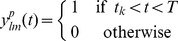
(9)


### Input, Initial and Boundary Conditions

The input to the network is a constant depolarizing current inserted into a central 4×4 cluster of excitatory neurons. This input emulates an epileptic focus. The neuronal membrane potentials were initialised to 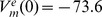 mV and 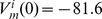 mV. The probability that transmitter was released from a presynaptic terminal was initialised to 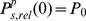. The remaining conductances and synaptic probabilities were initially set to zero for all simulations. Zero-boundary conditions were employed in the network so that the waves could not propagate back around the network and interfere with velocity calculations.

### Average Propagation Velocity Calculation

To determine the speeds of seizure propagation we considered 4 radial lines from the centre of the network and recorded the time and position of spiking neurons along these radial lines. The average velocity over the four lines is then calculated.

### Approximate Local Field Potential (LFP) Estimation

To determine if the network simulations produce the temporal frequencies observed in the EEG or local field potentials (LFPs) of real seizures, we calculated the simulated LFP, 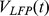, by averaging the membrane potentials of all the neurons in the network [12]:
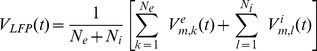
(10)where 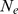 and 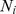 are the total number of excitatory and inhibitory neurons in the network, respectively, and 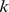 and 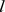 index the neurons in the excitatory and inhibitory populations, respectively. This LFP signal is simply a linear combination of the voltages of the neurons in the network and is intended to represent an approximation to an LFP recording from a small scale electrode close to the network, as opposed to a larger scale electrode that might be used in intra-cranial or scalp EEG in humans.

### Parameter Selection

Given the large number of parameters in the computational model it was not possible to exhaustively explore the parameter space, and instead we have adapted parameters from the Ursino and La Cara model [12] and from the literature.

Cortical excitatory and inhibitory neurons were modelled separately using intracellular parameters from regular spiking and fast spiking neurons, respectively [41], [42], [54], [55]. The parameters that characterise the excitatory and inhibitory neurons cellular membrane's: the membrane capacitances 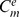, 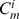, the leakage conductances 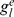, 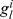, the reverse leakage potentials 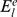, 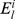, the reset potential 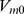, the spike generation threshold 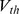, the duration of a spike 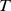 and the absolute refractory periods 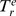, 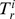 were given values commonly used in the literature [41], [42], [56]. The effective reversal potentials for the excitatory and inhibitory synapses 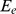 and 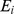 were also given values used extensively in the literature [12], [41], [42], [56].

The value for the maximal excitatory synaptic conductance, 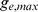 was selected based on the work carried out by Prinz *et al* [57]. Using IAF neurons Prinz observed that synaptic conductances in the range 10–100 nS have the greatest effect on the firing patterns of a neuron with values larger than 100 nS having the same effect. Hence, the maximal synaptic conductance was chosen to be 80 nS. The maximal inhibitory synaptic conductance 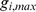 was taken to be larger than 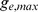 according to the literature [41], [42], [56]. This reasoning was used by Ursino and La Cara [12].

The parameters describing the dynamic behaviour of the synapses are 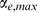, 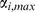, 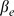 and 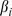 and have been selected based on values found in the literature [12], [42], [56].

The parameter, 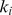, which determines the rate of saturation of the inhibitory synaptic conductance has been taken to be 4 times greater than 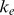 as the inhibitory cells are considered to respond faster than the excitatory cells. The size of the network was also chosen to reflect this fact with there being 2500 excitatory neurons and only 625 inhibitory neurons.

To select the values of the synaptic weights under ‘normal’ conditions the following ideas were considered. When a single pre-synaptic neuron fires the maximal change in 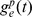, the excitatory conductance of a single post-synaptic neuron, is of the order of 3 nS [41]. In the model of Ursino and La Cara [12] this maximal change occurs when the excitatory synaptic strength equals 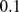. The corresponding maximal change in the inhibitory conductance 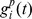 is of the order of 5 nS [41] and to achieve this change Ursino and La Cara [12] set the inhibitory synaptic strength to 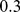. However, if the synaptic weights were kept at these values then nothing interesting occurs. To ensure that activity spreads beyond the focus when inhibition fails the synaptic weights are increased but kept in the same ratio of 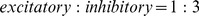. With these considerations in mind, when inhibition was broader than excitation, the following values were selected 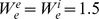 and 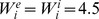.

Values for the standard deviations 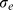 and 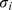 that govern the spatial extent over which inputs can act were determined by balancing the average current flowing into a single centrally located neuron assuming every neuron connected to it is spiking. This means the sum of the average current due to the excitatory synapses 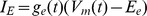 and the average current due to the inhibitory synapses 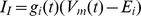 must be equal to zero. This condition results in an infinite number of choices for 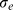 and 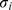, so keeping in mind that we are interested in parameter spaces where activity spreads beyond the focus when inhibition fails the following values were selected 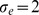 and 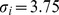.

For the case when excitation is broader than inhibition we set 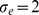 and 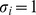. Since 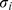 has now decreased, to achieve the same excitatory inhibitory balance as before the excitability of the inhibitory neurons must increase, the ability for excitatory neurons to be inhibited must increase and the ability for inhibitory neurons to be inhibited must decrease. Thus, the synaptic weights were adjusted as follows: 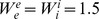, 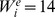 and 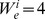.

The values for the parameters governing the relative refractory period and spike rate adaptation phenomena 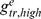, 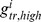, 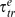, 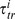 and 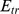 were chosen according to the literature [12].

Heiss *et al* [58] found that inhibition adapts more than excitation in rat cortex. The presynaptic depression parameters 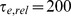
ms, 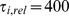
ms, 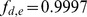 and 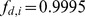 were selected so that the inhibitory and excitatory synaptic conductances on both the inhibitory and excitatory neurons adapted at a rate that corresponded to the experimental results of Heiss.

The model parameters under ‘normal’ conditions where activity does not spread beyond the focus as outlined above are summarised in Table 1.

**Table 1:** Model Parameters.

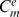	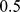 nF	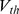	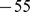 mV	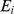	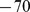 mV	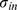	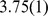	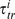	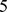 ms
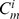	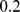 nF	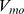	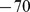 mV	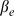	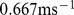	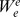	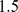	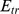	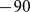 mV
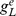	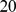 nS	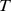	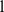 ms	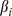	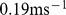	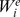	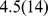	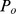	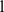
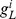	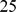 nS	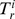	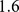 ms	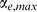	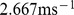	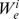	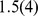	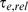	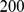 ms
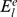	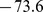 mV	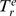	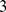 ms	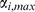	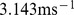	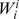	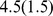	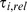	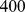 ms
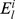	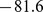 mV	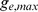	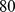 nS	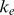	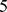	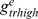	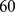 nS	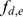	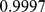
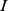	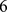 nA	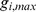	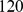 nS	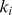	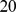	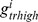	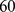 nS	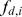	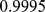
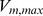	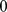 mV	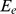	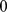 mV	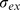	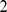	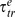	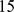 ms		

Computational model parameters under normal conditions where activity does not spread beyond focus. The values in brackets are used when excitation is broader than inhibition.

#### Low extracellular 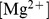 simulations

Under low extracellular 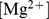 conditions the number of open NMDA receptors increases [59], [60]. As a result a number of parameter modifications need to be made with respect to the ‘normal’ case.

The first is that pre-synaptic effects on excitatory (NMDA) receptors cause a decrease in the rate of pre-synaptic adaptation of excitatory synapses. There are presynaptic NMDA receptors in cerebral cortex [28] and entorhinal cortex [61], [62]. In general, MacDermott [63] claims that presynaptic terminals of excitatory synapses have NMDA receptors, but the presynaptic terminals of inhibitory synapses typically do not. NMDA receptors pump Ca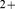 into the cell which was confirmed presynaptically by Woodhall [61]. Residual presynaptic Ca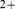 modulates presynaptic facilitation and depression as modeled by Dittman [64]. In this model recovery from depression results from an increase in residual Ca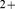 concentration. In the low extracellular 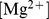 model we propose that there is an increase in the opening of the presynaptic NMDA receptors causing Ca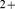 to go into the cell raising the Ca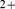 concentration which increases the rate of recovery from depression. As a result, in our simulations of the the low extracellular 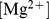 model, the parameters 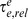 and 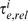 which control the rate of recovery from depression for excitatory synapses decrease in value from 200 ms to 100 ms. This change results in an overall increase in the rate of recovery from depression.

The second is that post-synaptic effects on excitatory (NMDA) receptors increase post-synaptic excitatory conductance [59], [60] due to the increase in the number of open NMDA receptors. To approximate this the parameter 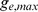 in the computational model increases from 80 nS to 90 nS.

Third, we propose that the cells in the inhibitory surround initially have enough activity to prevent the spread of excitatory activity but the inhibitory synapses adapt faster than excitatory synapses [58] and excitation eventually spreads. As a result we are interested in varying the parameters 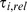 and 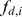 since they control the rate at which the inhibitory synapses are depressed in the computational model and hence govern the speed at which a seizure spreads.

We also considered the possibility that a change in the inhibitory reversal potential, 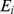, could lead to slow wave seizures, as changes in the GABA/chloride reversal potential [65] appear to play a role in the inhibitory restraint in low extracellular 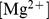 slices [66]. To simulate this case, the same parameter values as those described for the low extracellular 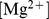 model were used except 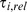 and 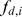 were fixed at ‘normal’ values and 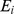 was varied, with fixed values in each simulation.

#### GABA antagonist simulations

For the GABA antagonist model simulations we assume GABA antagonists are present. Therefore inhibition is either weakened or removed completely. We achieve this in simulations by reducing the inhibitory synaptic weights, 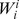 and 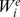 below their normal values.

### Numerical Simulation and Spatial Analysis

The numerical method used to solve the differential equations in all of our simulations is the fourth-order Runge-Kutta method with a 0.1 ms time step.

To quantify the spatial characteristics of the waves in the network we determined the Mean Correlation Coefficient (MCC), also referred to as spatial coherence [67]. To calculate the MCC we determine the correlation coefficient for each neuron with every other neuron in a 200 ms window. This generates a 2500×2500 array of correlation coefficients for the excitatory network and a 625×625 array for the inhibitory network. The mean of the correlation coefficients for each type of network is then calculated excluding the autocorrelation values. To produce a time series of MCC's the same procedure is carried out at 5 ms time steps, that is the windows have a 195 ms overlap. In all our MCC results figures we only include the excitatory network since the MCC of the inhibitory network behaves in a similar way and therefore delivers no extra insight.

## Results

The suggested model has many parameters that could be varied, creating an impossible number of combinations to analyse. For this reason, we focus on the parameters associated with the synaptic weights and the synaptic connectivity for both slice model simulations as well as the presynaptic depression parameters for inhibitory neurons and the inhibitory reversal potential in the low extracellular 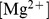 simulations. Given this approach it should be stated that the dynamical properties observed in this paper may correspond to only a subset of possible behaviours of this model. Nevertheless, the results provide insight into the mechanisms of seizure propagation and the parameters which we found propagation velocity was most sensitive to.

### GABA Antagonist Model Simulations

The simulations performed in this section use parameter values as described for the GABA antagonist model.

#### Fast temporal frequency correlates with fast wave propagation

**Figure 1:**
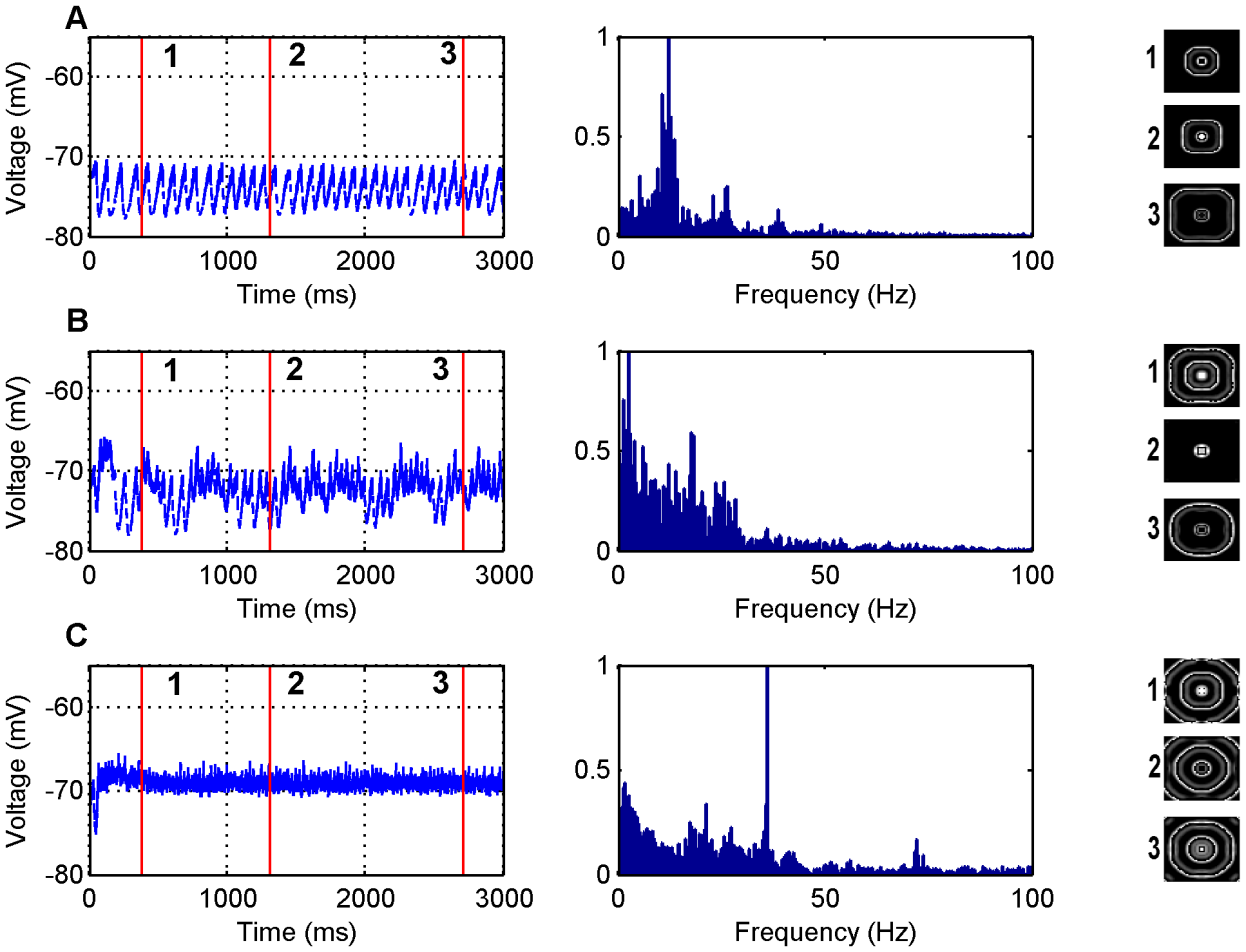
LFP simulations over a 3 second interval for the GABA antagonist model for various excitatory synaptic weights where the inhibitory weights have been set to zero. In each row the simulated LFP in the time (left) and frequency (middle) domains are presented, along with snapshots of network activity (right) at different time steps during the simulation (1) 384 ms, (2) 1308 ms and (3) 2708 ms. The snapshots are of the excitatory network only. The parameter values used are: (A) 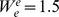, (B) 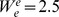, (C) 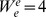.

The first set of simulations performed as shown in Figure 1 detail the LFP of seizure-like events in both the time and frequency domains for various values of the excitatory synaptic weight 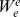 when the inhibitory weights are set to zero. When the excitatory synaptic weight is at its ‘normal’ value, 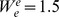, as is the case in panel A, circular waves propagating away from the central focus can be observed. The frequency content of these waves is concentrated in the 0–15 Hz range. As the excitatory synaptic weight increases to 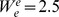 as in panel B, circular waves continue to propagate away from the central focus but at a faster rate. This results in the frequency content of the wave occupying a broader range from 0–30 Hz. When the excitatory synaptic weight increases to an even larger value 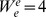 as in panel C a very sharp frequency peak is observed at 36 Hz. These frequencies are consistent with seizure frequencies of 3–48 Hz that are typically observed physiologically in humans [68], [69]. There is limited data on the power spectra of LFPs recorded from disinhibition slices. In an *in vivo* rat study of disinhibition [70], it was observed that frequencies in the range of 5–20 Hz occurred prior to clinical seizures. Intuitively, as long as the spatial scale of interaction remains approximately constant, it is expected that the faster the seizure propagation velocity the faster the LFP frequency. Therefore disinhibition slices would be expected to be biased towards higher LFP frequencies.

#### No inhibition: excitatory strengths effect correlation patterns

**Figure 2:**
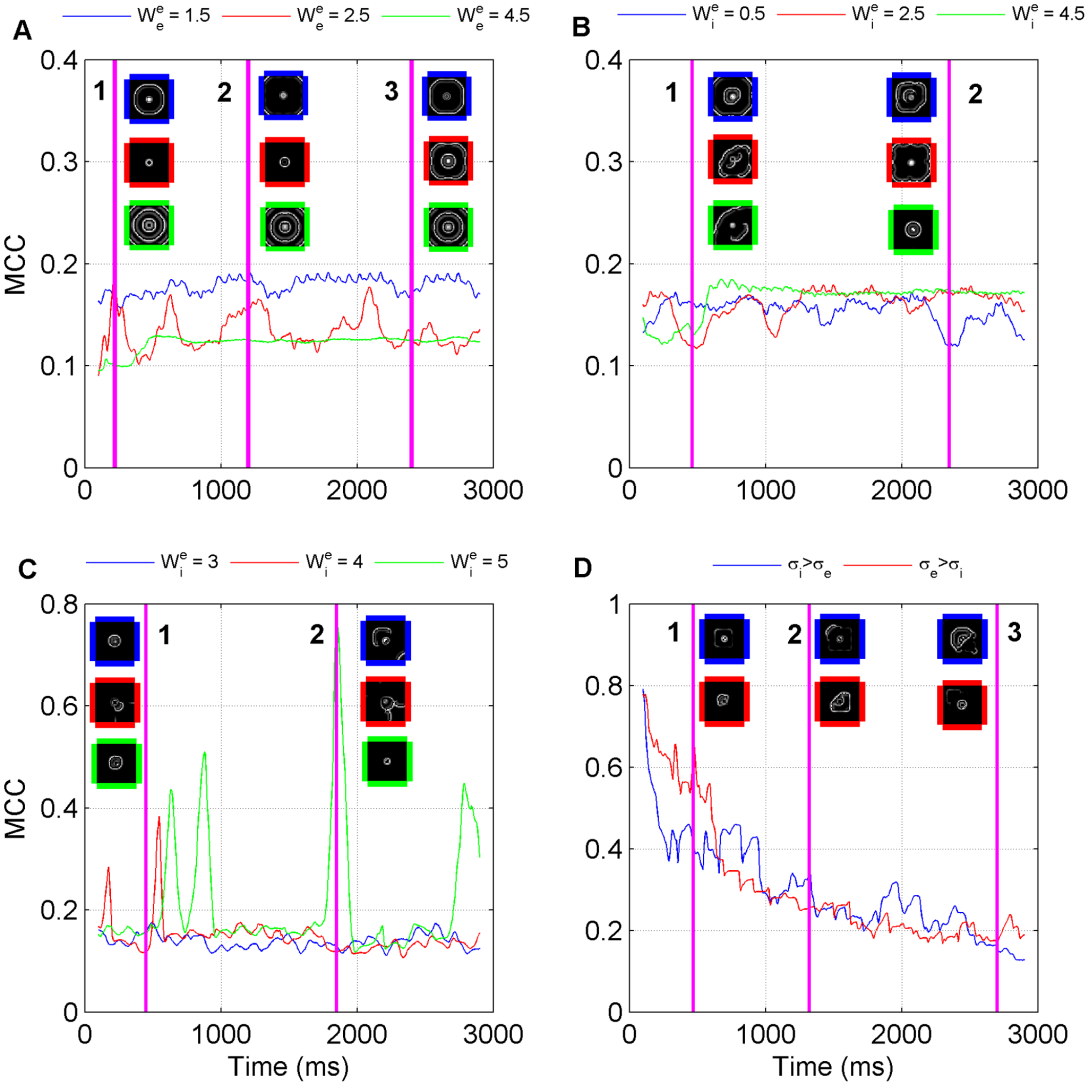
Mean Correlation Coefficient spatial analysis for the various simulations. (A) The GABA antagonist model for various excitatory synaptic weights where the inhibitory weights have been set to zero. The snapshots correspond to excitatory network activity at times (1) 220 ms (2) 1200 ms and (3) 2400 ms. (B) The GABA antagonist model for various excitatory synaptic weights where the inhibitory weights are non-zero and 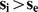. The snapshots correspond to excitatory network activity at times (1) 460 ms and (2) 2350 ms. (C) The GABA antagonist model for various excitatory synaptic weights where the inhibitory weights are non-zero and 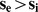. The snapshots correspond to excitatory network activity at times (1) 450 ms and (2) 1850 ms. (D) The low extracellular [Mg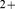] model for 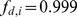 when 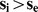 and for 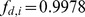 when 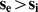. The snapshots correspond to excitatory network activity at times (1) 470 ms (2) 1320 ms and (3) 2700 ms.

Next, we are interested in quantifying the spatial characteristics of the waves and this is considered in Figure 2A which details the MCC for various values of the excitatory synaptic weight when the inhibitory weights are set to zero (Note the remainder of the panels in Figure 2 are discussed as each simulation case is considered). For 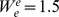 the MCC is high. This is due to there being a large number of inactive neurons in the network hence the correlation between them is large. However, as we increase 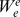 to 2.5 we observe fluctuations in the MCC. Under these conditions there are bursts of activity in the network with there being periods of time where lots of waves are being generated at high frequency which correspond to the low MCC periods and periods where few or no waves are being generated which corresponds to the high MCC periods. For the final case where 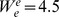 the MCC is constant after some initial transient with uniform waves being generated constantly over time. The network behaviour for each value of 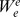 can be viewed in Videos S1, S2, and S3.

#### Varied inhibition with inhibition broader than excitation

**Figure 3:**
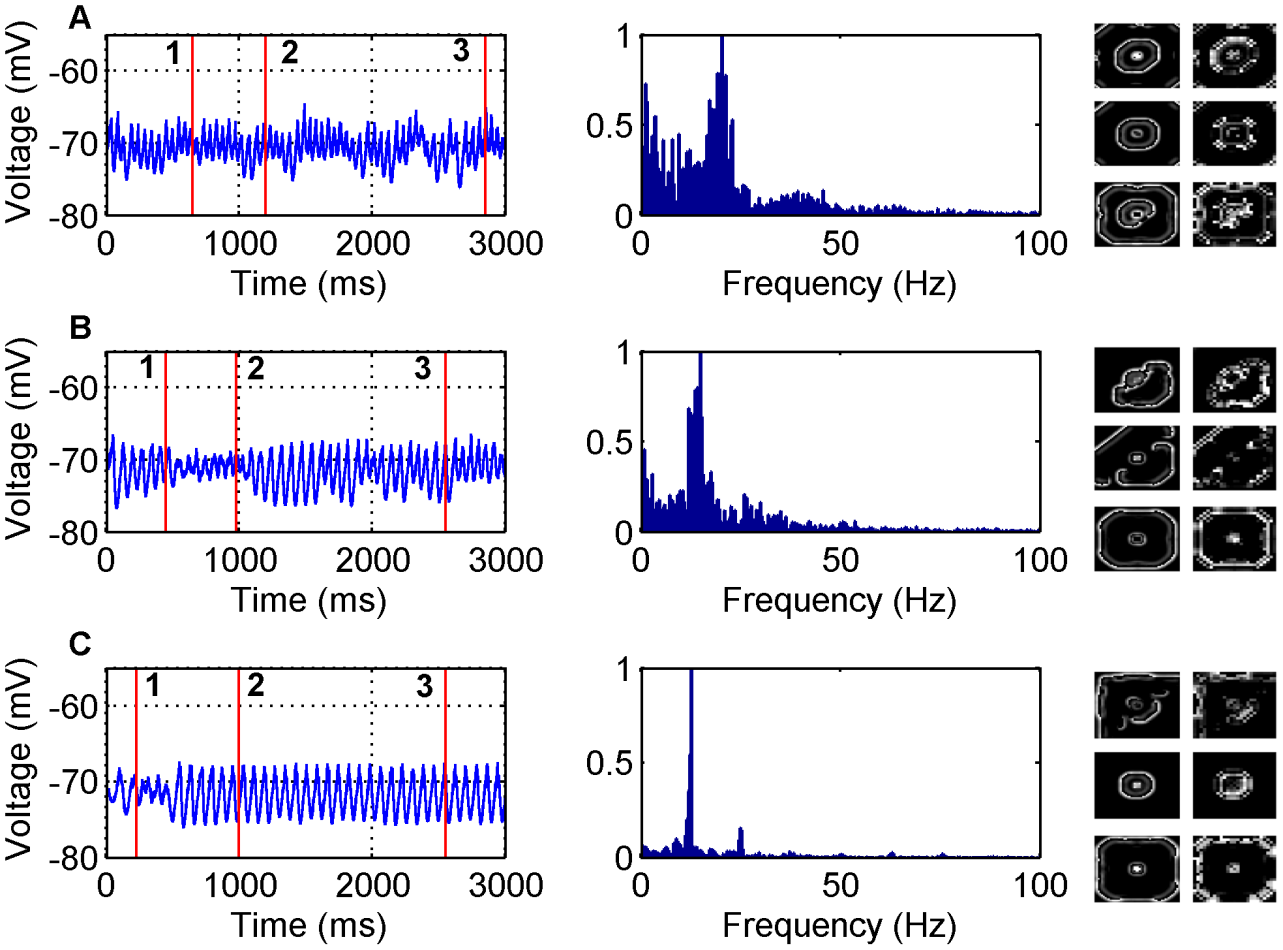
LFP simulations over a 3 second interval for the GABA antagonist model for various non-zero inhibitory synaptic weights and 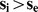. In each row the simulated LFP in the time (left) and frequency (middle) domains are presented, along with snapshots of network activity (right) at different time steps during the simulation (A) - (1) 650 ms, (2) 1200 ms (3) 2800 ms, (B) - (1) 450 ms, (2) 980 ms, (3) 2550 ms, (C) - (1) 230 ms, (2) 1000 ms, (3) 2550 ms. The snapshots include both the excitatory (left) and inhibitory (right) populations. The parameter values used are: (A) 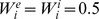, (B) 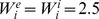, (C)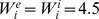. 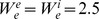.

To further understand the influences of inhibition on network dynamics and propagation velocity, simulations were carried out for the GABA antagonist model with varying degrees of inhibition. The first case considered as shown in Figures 3 and 2B is when 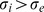. When the inhibitory weights are close to zero, 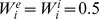, as is the case in Figure 3A, we see relatively circular waves propagating away from the central focus in both the inhibitory and excitatory networks. The frequency content of the waves are in the range 0–30 Hz. As the inhibitory weights are increased to 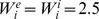 as shown in Figure 3B the frequency content becomes narrower and centres around the 15 Hz mark. When the inhibitory weights are increased even further to 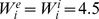 as shown in Figure 3C the frequency content is more concentrated with a sharp peak observed at 12 Hz.

Figure 2B indicates that for 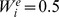 relatively circular waves propagate for the first 2200 ms but when there is a sharp drop in the MCC more disordered waves occur. For 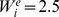 when there is a sharp dip in the MCC at 300 ms disordered waves occur, then the MCC begins to rise again at 600 ms where the waves are highly non-circular but are occurring regularly. After 1250 ms the disordered waves cease and relatively circular waves begin to propagate. However, every time there is a decrease in the MCC the waves become more disordered. When 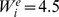 similar behaviour occurs with disordered waves occuring when the MCC is low for the first 600 ms after which the MCC increases to a steady value where relatively circular waves propagate. The network behaviour for each value of 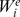 can be viewed in Videos S4, S5, and S6.

#### Varied inhibition with excitation broader than inhibition

**Figure 4:**
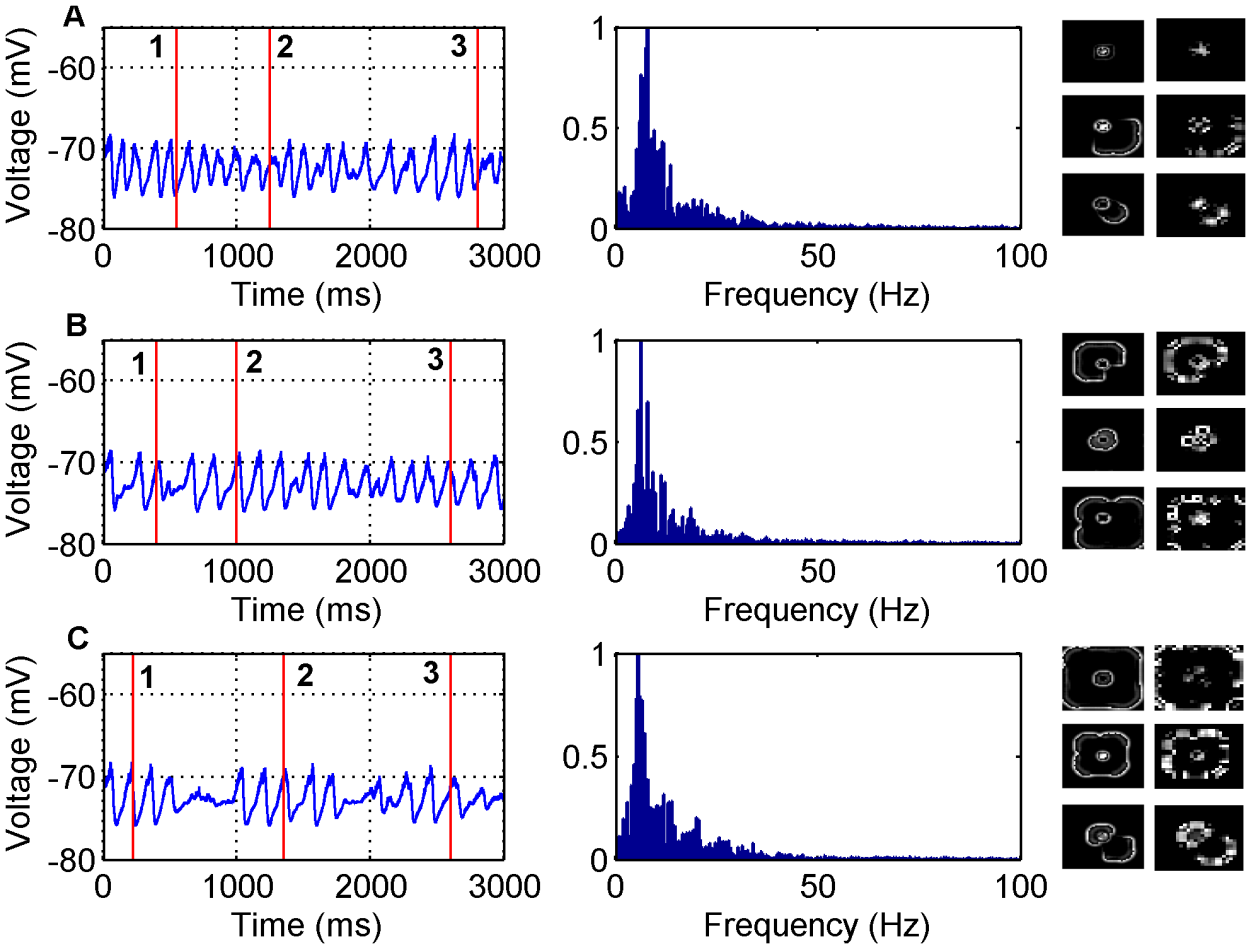
LFP simulations over a 3 second interval for the GABA antagonist model for various non-zero inhibitory synaptic weights and 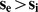. In each row the simulated LFP in the time (left) and frequency (middle) domains are presented, along with snapshots of network activity (right) at different time steps during the simulation (A) - (1) 550 ms, (2) 1250 ms (3) 2800 ms, (B) - (1) 400 ms, (2) 1000 ms, (3) 2600 ms, (C) - (1) 225 ms, (2) 1350 ms, (3) 2600 ms. The snapshots include both the excitatory (left) and inhibitory (right) populations. The parameter values used are: (A) 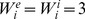, (B) 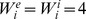, (C)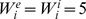.

The second GABA antagonist model with non-zero inhibition case considered is shown in Figures 4 and 2C where 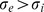. Figure 4A shows that when 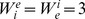 the frequency content is concentrated in the 0–20 Hz range with a peak at 8 Hz. Figure 4B shows that when 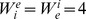 the frequency content is still within the 0–25 Hz range but now has a peak at 6.5 Hz. Figure 4C shows that when 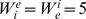 the frequency content is again within the 0–25 Hz range but with a peak that has shifted to 5.5 Hz. Note that the 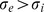 case produces lower peak temporal frequencies than the 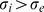 case.

The network snapshots along with Figure 2C indicates that the waves are disordered. We also notice that as we increase the inhibitory weights there are large spikes in the MCC. This corresponds to times when there is little or no network activity. This is expected since as inhibition gets stronger there are more occasions where neurons are not spiking. The network behaviour for each value of 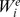 can be viewed in Videos S7, S8, and S9.

#### Increased inhibition increases wave disorder

The data from Figures 1, 2, 3, 4 also indicate that how disordered the waves are is dependent on the inhibitory neurons. When there is zero inhibition present circular waves occur, when a small amount of inhibition is allowed the waves become less circular and when the amount of inhibition increases further, completely disordered waves ensue. One reason the inhibitory neurons play such a role in the disorder of the waves is due to the topology of the network. Essentially there is a 50×50 layer of excitatory neurons arranged in a regular lattice but there is only a 25×25 layer of inhibitory neurons. So that the excitatory and inhibitory neurons occupy the same space there is only an inhibitory neuron at every second location in the 50×50 lattice. This creates an asymmetry relative to the input seizure focus and thus more disordered waves are observed when the inhibitory neurons become more active (see discussion). However, we also observe that as inhibition becomes strong there are disordered waves for an initial period of time and then more circular waves propagate for the rest of the simulation. This effect is caused by the presynaptic adaptation where inhibition decreases due to the high amount of activity and so the reduction in inhibition results in more circular waves.

#### An effective threshold for activity propagation

**Figure 5:**
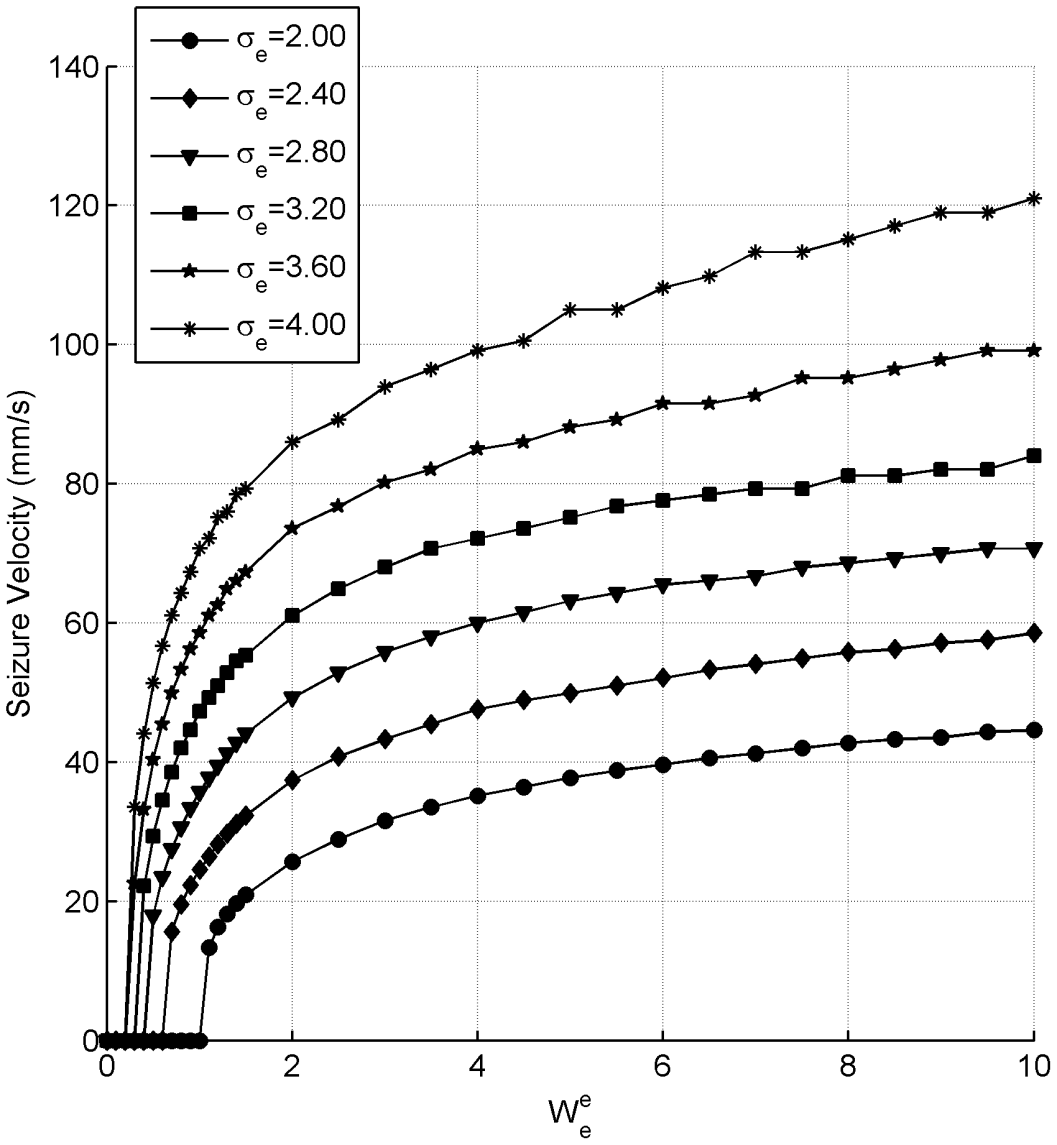
Seizure propagation speeds for the GABA antagonist simulations with zero inhibition. The impact that the extent of excitatory connectivity, 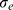, and the excitatory synaptic strength of the excitatory neurons, 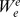, has on propagation speeds is considered.

The next set of simulations performed as shown in Figure 5 outline the speed at which the outwardly propagating circular waves move for different values of the excitatory synaptic strength 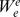 and the excitatory synaptic connectivity 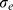, when there is zero inhibition. The first observation to make is that an increase in either the synaptic connectivity, 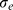 or the synaptic strength 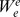 causes the propagation speed to increase. However, 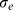 has a much greater impact on the propagation speed than the synaptic strength 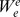. As 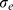 is increased the propagation speeds continue to increase without saturation. The maximum propagation speed achieved was 316 mm/s for a 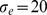. This suggests that the upper bound on the propagation speed is large and indeed can be large enough for the model to be physiologically plausible. The most significant observation to make, however, is that there are effective threshold values of 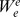 and 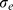 for seizure-like activity to occur. Consider the 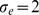 curve, the minimum value of the excitatory synaptic weight for seizure-like activity to occur is 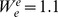. Anything below this value results in no activity spreading beyond the focus. For all cases considered in Figure 5, it can be noted that with zero-inhibition, clean circular wavefronts are produced with fast propagation speeds that have an effective threshold between activity spreading and not spreading which involves a very narrow range of parameter values over which velocities increase from 0 to greater than 10 mm/s.

**Figure 6:**
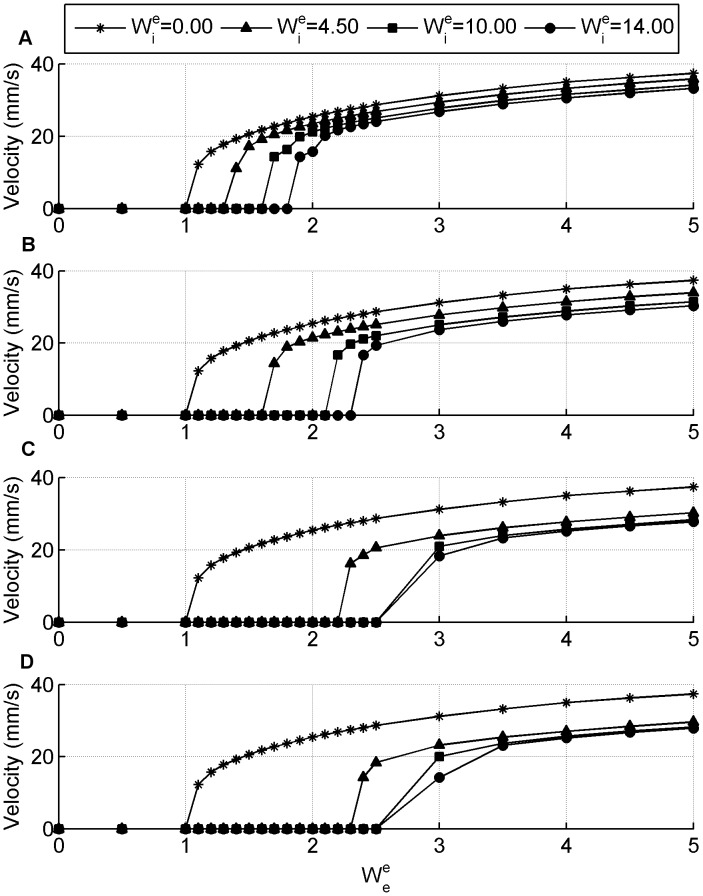
Seizure propagation speeds for the GABA antagonist simulations with non-zero inhibition. The impact that the inhibitory synaptic weights 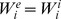 and the excitatory synaptic weights 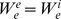 have on propagation speeds is considered for different values of inhibitory extension (A) 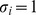, (B) 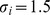, (C) 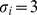, (D) 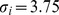. The excitatory connectivity is fixed at 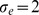.

Since our computational model contains a separate inhibitory population it is of interest to examine how the degree of inhibition effects the seizure propagation speeds. Figure 6 looks at how changing the synaptic weights 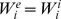, 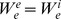 and the synaptic connectivity 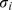 for a fixed 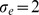 effects propagation speed. All panels contain a reference curve of the zero inhibition case 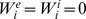. The first observation to make is that all panels show that by including inhibition the propagation speed is smaller than in the zero-inhibition case. However, again the most significant observation to make is that there are effectively two operating regions, there is either no spread of activity occurring or there is activity but primarily with speeds greater than 15 mm/s. There is only a small dynamic range for 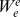 over which the velocity increases from 0 mm/s to greater than 10 mm/s.

#### GABA antagonist simulation summary

The typical behaviour for the GABA antagonist model is that it can produce both ordered and disordered waves depending on the strength of inhibition in the network and that there is always an effective threshold on the balance of excitatory and inhibitory input between activity spreading and not spreading. This balance between excitation and inhibition is reflected in the excitatory and inhibitory strengths as shown in Figures 5 and 6, where the effective threshold is characterised in part by the very narrow range of excitatory connection strength values over which velocities change from 0 to 10–20 mm/s. Thus for the range of parameters considered, simulated activity is either most likely not to propagate or to propagate at velocities in the 10–100 mm/s range. This is consistent with the literature on GABA antagonists in brain slices [33], [34].

### Low Extracellular 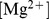 Model Simulations

The simulations performed in this section use parameter values as described for the low extracellular 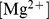 model.

#### Temporal frequencies are higher for excitation broader than inhibition

**Figure 7:**
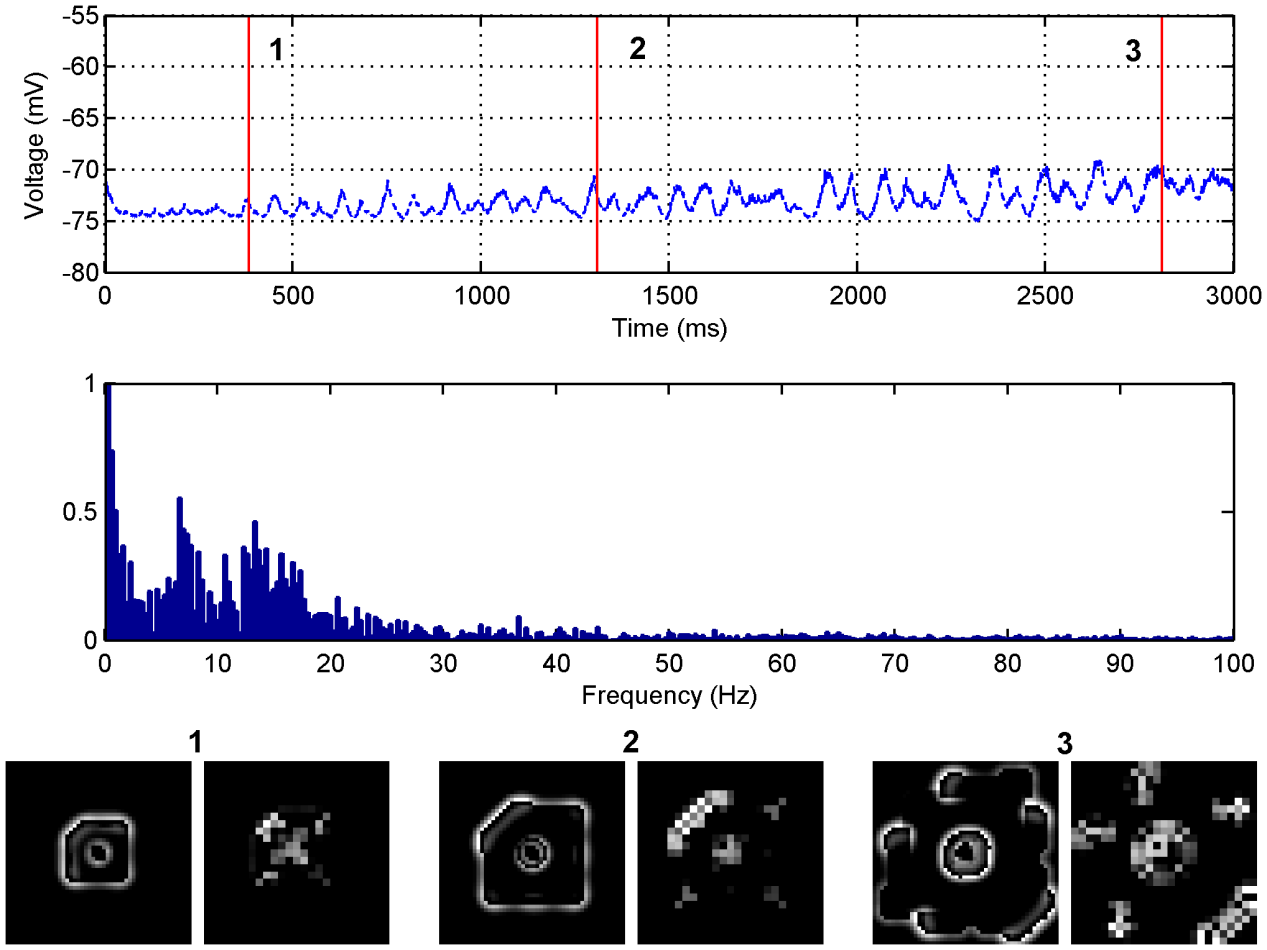
LFP simulations over a 3 second interval for the low extracellular [Mg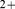] model with 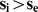. The simulated LFP in the time (top) and frequency (middle) domains are presented, along with snapshots of network activity (bottom) at different time steps during the simulation (1) 384 ms, (2) 1308 ms and (3) 2808 ms. Each snapshot contains the excitatory (left) and inhibitory (right) networks. The presynaptic depression factor for the inhibitory neurons is 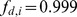.

**Figure 8:**
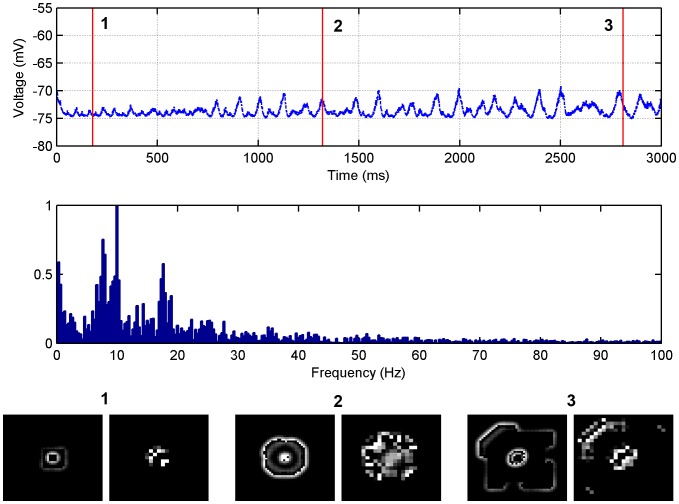
LFP simulations over a 3 second interval for the low extracellular [Mg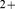] model with 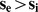. The simulated LFP in the time (top) and frequency (middle) domains are presented, along with snapshots of network activity (bottom) at different time steps during the simulation (1) 384 ms, (2) 1308 ms and (3) 2808 ms. Each snapshot contains the excitatory (left) and inhibitory (right) networks. The presynaptic depression factor for the inhibitory neurons is 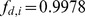.

The first set of simulations performed for this model as shown in Figure 7 when 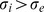 and in Figure 8 when 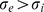, detail the LFP of a seizure-like event in both the time and frequency domains for a particular value of the presynaptic depression factor for the inhibitory neurons 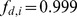 and 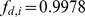, respectively. For both figure's snapshots 1–3, all depict the same wavefront at different points in time and they indicate that the wave is propagating slowly away from the central focus with both cases being disordered. For both cases the amplitude of the LFP in the time domain slowly increases as time progresses due to more neurons firing as the wave propagates. The frequency content of the wave in Figure 7 is within the 0–20 Hz range. In Figure 8 the frequency content is more concentrated and with a peak at 10 Hz, however, the majority of the content lies within the 0–30 Hz range. Again, this is consistent with seizure frequencies of 3–48 Hz that are typically observed physiologically in humans [68], [69]. There is also limited data on the LFP power spectral content for low extracellular 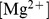 slices. Trevelyan [66] looked at high frequency oscillations in the range of 80–500 Hz in low extracellular 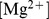 slices, however, such frequencies, although important and present, are not expected to be the dominant frequencies in the LFP signal on average. In a somewhat similar *in vivo* model of sleep-like slow-waves which produces slow velocity waves [67], it was found that the spatial average of voltage-dye imaging signals predominantly contained temporal frequencies in the range of 0–20 Hz. Videos of the simulations presented in Figures 7 and 8 are available as Videos S10 and S11, respectively.

#### Spatial Correlation Decreases as Activity Spreads

Figure 2D depicts the MCC for the low extracellular magnesium case for both 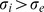 and 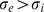. The MCC starts off large indicating little or no network activity and then decays over time highlighting the fact that disordered activity in the network is spreading slowly over time and this leads to greater spatial decorrelation across the network over time.

#### Inhibitory Depression Controls Slow Propagation

**Figure 9:**
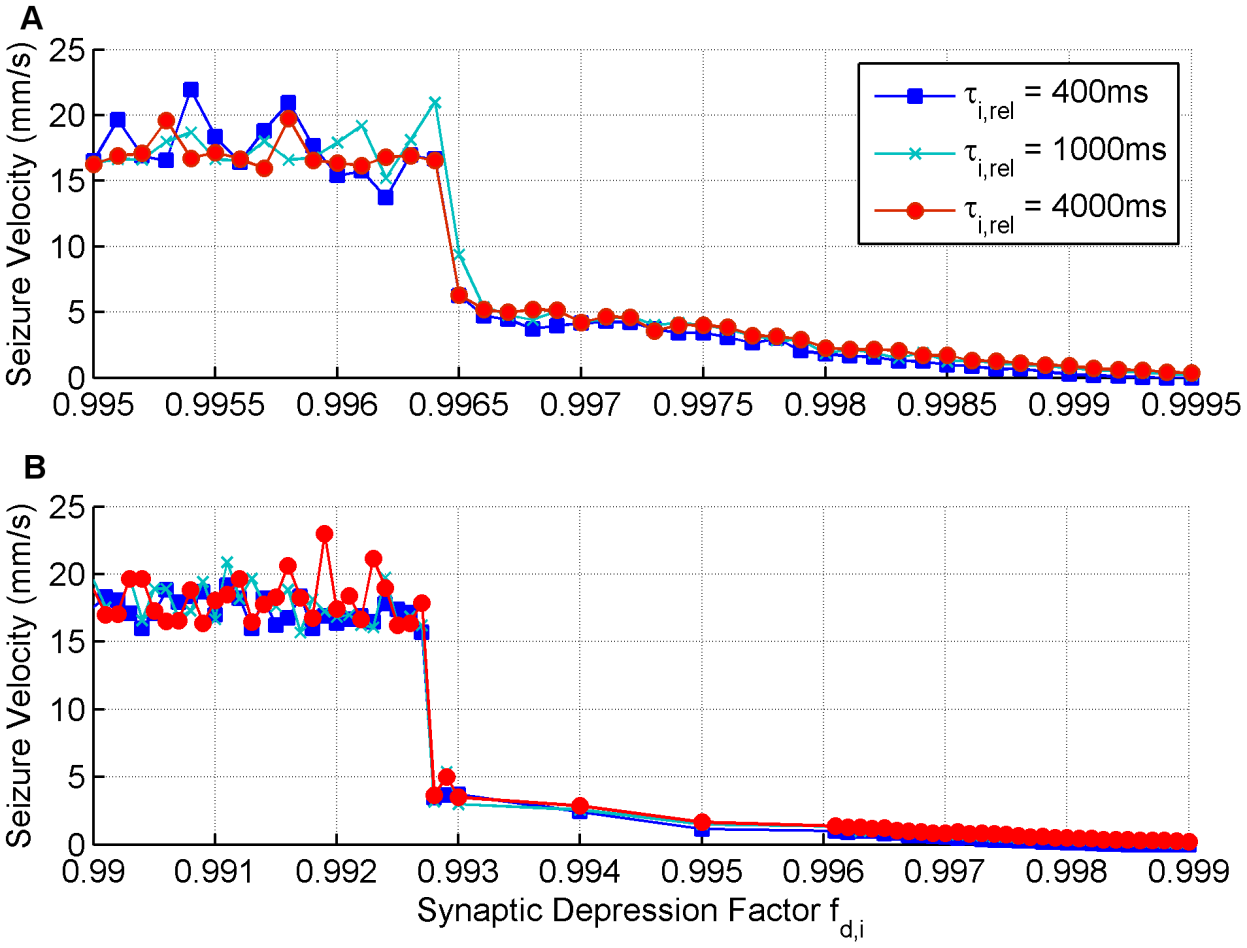
Seizure propagation speeds for the low extracellular [Mg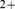] simulations. The impact that the inhibitory presynaptic depression factor and the inhibitory presynaptic depression recovery time has on propagation speeds is considered when (A) 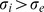 and when (B) 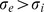.

The next set of simulations performed as shown in Figure 9 outline the speed at which the wavefront moves for different values of the inhibitory presynaptic depression factor 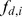 and the inhibitory presynaptic depression recovery time 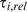. The first observation to make, is that in both cases, the recovery time 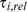 has little impact on the propagation speed of the wavefront, even when it is increased to a large number so as to slow the recovery of the adapted presynaptic terminal. On the other hand, the depression factor 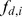 has a significant effect. Other parameters were also varied and network activity was simulated (results not shown), but the key parameter needed to create slow seizure propagation speeds was the presynaptic inhibitory depression factor, 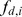. The next observation to make is that there are two distinct trends in the propagation speeds.

For small inhibitory presynaptic depression factors, inhibition is suppressed quickly, so seizures spread as if there is no inhibition at all, hence the speed of propagation saturates to between 15–20 mm/s. When the presynaptic depression factor reaches a threshold, 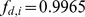 for the 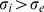 case and 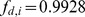 for the 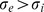 case, the inhibition is suppressed at a rate slow enough that propagation speeds can be controlled and very slow speeds can be attained. The inhibitory presynaptic depression factor threshold effect emerges because for a low enough inhibitory presynaptic depression factor the temporal summation of incoming spikes will cause the probability of presynaptic release at inhibitory synapses to go to zero (see equation 4) thus approximating the GABA antagonist model with zero inhibition and giving higher velocities. Because spike arrival is noisy the velocity estimates also end up noisy for different simulations, thus giving rise to the fluctuations seen in the plateau of velocity values in the range of 15–20 mm/s for different inhibitory presynaptic depression factor values below a certain threshold. When we compare the two connectivity kernel cases we notice that the same behaviour is observed but over a different range of the presynaptic depression factor with slower speeds being possible over a greater range of 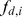 for the case when 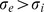. To summarise, disordered wavefronts (generally observed in all low 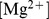 simulations) combined with inhibition that depresses faster than excitation result in slow propagation speeds, and there is an effective threshold between activity spreading slowly and it spreading at a rate similar to that observed in the GABA antagonist simulations when inhibition becomes fully depressed.

### Simulation of the Jacksonian March

**Figure 10:**
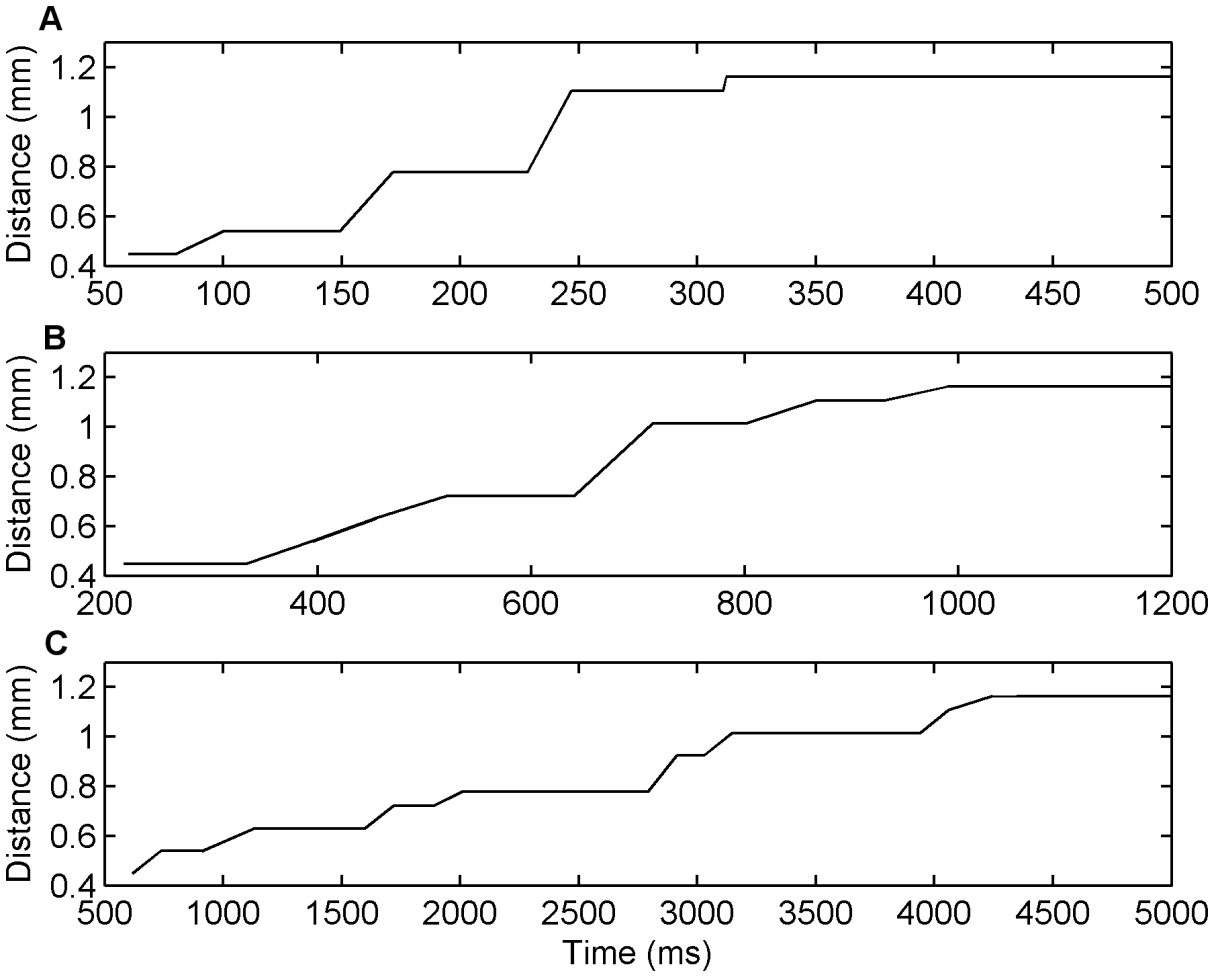
Jacksonian March propagation patterns in the low extracellular [Mg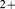] simulations for seizure velocities of (A) 3.69 mm/s, (B) 1.0 mm/s, and (C) 0.17 mm/s. In each plot, the y-axis indicates the distance (mm) that network activity has travelled within a certain period of time, where distance is defined radially relative to the centre of the network. The x-axis indicates time (s).

An interesting behaviour of the low extracellular 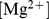 animal slice model is its ability to produce seizure-like activity that progresses from one region to another in a step-like manner as opposed to a smoothly propagating wavefront. This behaviour is reminiscent of the ‘Jacksonian March’ observed in tonic-clonic seizures in humans [32]. Figure 10 shows this behaviour for the low extracellular 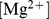 simulations for the propagation velocities of (A) 3.69 mm/s, (B) 1.0 mm/s, and (C) 0.17 mm/s with each plot indicating how far away the wavefront is from the centre of the network at any instant in time. In all three plots the stepwise movement of the propagating wavefront is evident over the different time scales.

#### Low extracellular 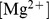 simulation summary

The typical behaviour for the low extracellular 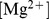 simulations is that there is an effective threshold on the inhibitory presynaptic depression factor between activity propagating slowly and quickly, and the slower the wavefront propagates the more stepwise the propagation. This slow, staggered propagation results from a progressive weakening of inhibition across the network via inhibitory presynaptic depression occurring faster than excitatory presynaptic depression. The slow propagation speeds are of the order of 0.1–10 mm/s which is within the lower range for seizure speeds as outlined in the literature, and corresponds closely to the range of speeds observed for low extracellular 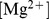 slices [22], [23], [27].

#### The influence of the inhibitory reversal potential

As mentioned in the methods, we considered whether or not slow velocity seizures could be obtained for the low extracellular 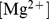 model if we held the inhibitory presynaptic depression parameters at ‘normal’ values and varied the inhibitory reversal potential, 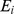, with fixed values for each simulation. It was found that making 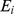 more positive had a similar effect as decreasing the inhibitory synaptic strength in the GABA antagonist model: first no seizures were observed and then seizures began to emerge with velocities rapidly increasing to around 10–14 mm/s or faster (figure not included). At the sharp velocity transition point slower velocities could be obtained for a very narrow range of 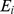 values. For 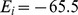 mV the seizure velocity was 0 mm/s. This increased exponentially to around 16 mm/s as 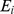 was increased to −65.1 mV, after which velocities increased at a slower than linear rate. However, when presynaptic depression of both excitatory and inhibitory synapses was removed from the model, it was no longer possible to obtain propagation velocities less than 10 mm/s. Moreover, when presynaptic depression was included the slow waves obtained are still more disordered and propagation is less like a ‘Jacksonian march’ than is observed for the low extracellular 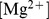 simulations involving changes in the inhibitory synaptic depression factor.

### Minimal Perturbation for Slow Seizure Propagation

We explored the minimal parameter perturbation of the ‘normal’ parameter values necessary to create slow velocity ‘Jacksonian March’ type seizure. This is done for two cases, the first is when 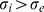 and the second is when 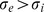. This is of interest because one would like to know the simplest change needed to allow seizures to propagate. Such simple changes may actually occur in ‘normal’ tissue when it is bombarded by activity coming from a seizure focus. In the low extracellular 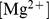 and GABA antagonist simulations the entire network in the slice is effectively abnormal/epileptic and therefore they do not provide the best examples of how seizures can spread through normal tissue.

**Figure 11:**
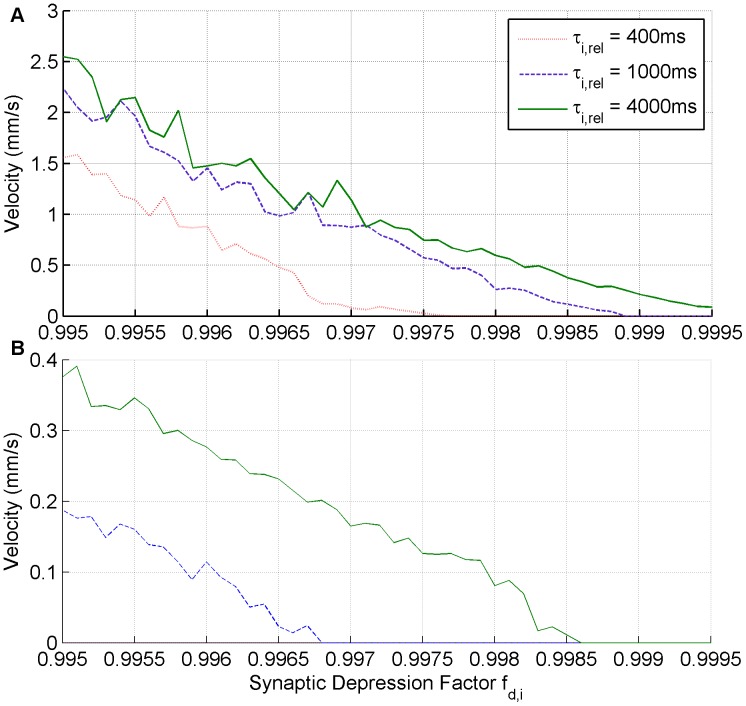
Minimal perturbation from ‘normal’ required for slow seizure propagation. The impact that the inhibitory presynaptic depression factor and the inhibitory presynaptic depression recovery time has on propagation speeds is considered when (A) 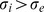 and when (B) 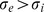.

Through exploration it was found that the most sensitive parameter that can produce slow velocity ‘Jacksonian March’ type seizure propagation when varied relative to the ‘normal’ parameter settings, is the inhibitory presynaptic depression factor 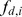. Figure 11 outlines the speed at which waves move for different values of the inhibitory presynaptic depression factor 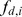 and the inhibitory presynaptic depression recovery time 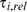. These waves have velocities that are slower for a given presynaptic depression factor when compared to the low extracellular 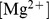 case. Moreover, as was the case with the low extracellular 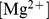 simulations, when 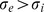 wave propagation is slower than when 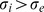.

### The Influence of Propagation Delays

**Figure 12:**
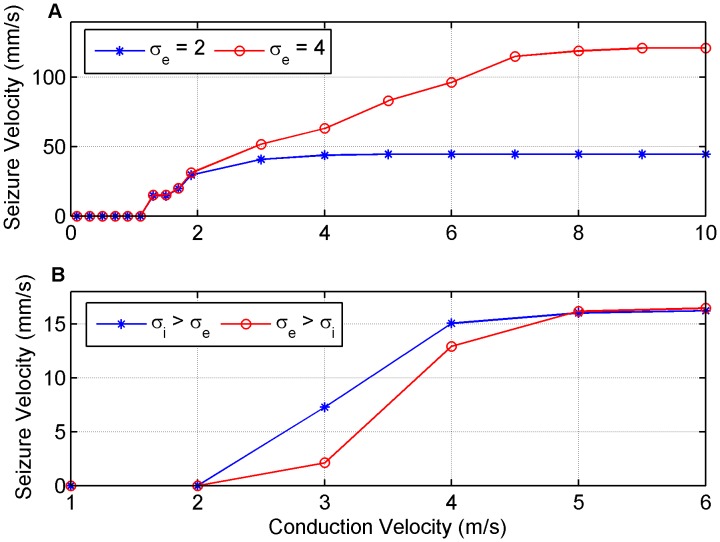
The effect axonal conduction velocity has on seizure propagation speeds in both the (A) GABA antagonist with zero inhibition and (B) low extracellular [Mg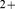] simulations. For the GABA antagonist case 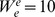 with the remaining parameters having ‘normal’ values. For the low extracellular [Mg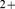] case 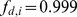 when 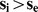 and 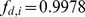 when 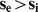.

In order to make the exploration of parameter space for both the GABA blocking and low extracellular 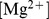 simulations more time efficient, the simulations up to this point have not included distance-dependent axonal propagation delays. To evaluate the potential effect axonal propagation delays would have on the network in the two slice simulations we consider Figure 12 where the delay is governed by the distance between two neurons as well as the conduction velocity of an action potential. Physiological conduction velocities have been estimated to be between 0.5–74 m/s [36], [71], [72] depending on whether the axon is myelinated. Considering the fastest seizure propagation speed of 120 mm/s seen in our figures for our computational model, the action potential velocity is between 4–720 times faster. By comparing Figure 12A where 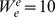 and 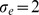 or 4 with the corresponding points on Figure 5, it can be noted that the inclusion of delays into the GABA antagonist model with zero inhibition did not effect the seizure velocity for conduction velocities greater than 4 m/s for the 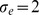 case and 8 m/s for the 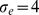 case. When the conduction velocities were less than these values seizure velocity reduced, however, the behaviour of the model is still preserved, such that there is an effective threshold velocity corresponding to whether or not activity spreads. The seizure velocity at which this threshold occurs also remains of the order of 10 mm/s. If we now compare Figure 12B where 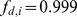 and 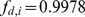 for the 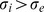 and 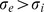 cases respectively, with the corresponding points on Figure 9 we again observe that by including delays into the low extracellular 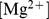 simulations, seizure velocity is unaffected if the conduction velocities are larger than 5 m/s. For conduction velocities below this the dynamical behaviour of the network is largely not effected, except for very low conduction velocities. In both the GABA antagonist and low extracellular 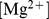 cases very low conduction velocities lead to zero seizure velocities as a result of the local network connectivities. When the conduction delays are longer, local coalesced neural activity becomes more spread out across time. This causes a reduction in the coincidence in neural activity and the ability for local groups to sustain larger scale activity which is needed for propagation of the wavefront to occur.

## Discussion

In this study we have examined the propagation of seizure-like activity using a computational model to simulate approximations of two *in vitro* slice preparations. We have illustrated how the full range of observed propagation velocities can be simulated with one and the same model under two different parameter sets that represent the two animal slice models. The first slice model considered was the GABA antagonist model; the simulations for which produced seizure propagation speeds of between 10–100 mm/s. This is within the upper range as outlined in the literature [22], [23], [33], [34]. The second slice model considered was the low extracellular 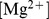 model, the simulations for which produced seizure propagation speeds of between 0.1–20 mm/s which is within the lower range as outlined in the literature [22], [23], [27]. In our simulations this range of velocities could be achieved when either excitation is broader than inhibition or inhibition is broader than excitation. For the GABA antagonist simulations, propagation was not necessarily faster when excitation was broader than inhibition, however, less excitation was needed in this case for waves to propagate.

Generally, for the GABA antagonist simulations it was observed that there is an effective threshold degree of inhibition (or balance of excitatory and inhibitory inputs/strengths) below which seizures begin to spread, which in part could explain why the empirical GABA antagonist slice model generally produces velocities above 10 mm/s. For the low extracellular 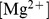 simulations, propagation was slower when excitation was broader than inhibition. The low extracellular 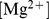 simulations also showed that seizure propagation occurred as a slow, stepwise recruitment of neurons in the computational network, akin to the ‘Jacksonian march’ seen in tonic-clonic seizures. The slow velocities and the ‘Jacksonian march’ primarily occur because when the inhibitory synaptic depression factor is reduced slightly, inhibition adapts faster than excitation allowing activity to spread. Since the synaptic depression is modelled as a dynamic process that acts slowly relative to neuronal spiking it takes several spike wavefronts to cause enough depression of inhibition to allow the activity to spread further out. This produces the slow, staggered propagation of the seizure. Finally, the computational model is able to simulate slow velocity ‘Jacksonian March’ type seizure propagation by only perturbing the inhibitory presynaptic depression factor relative to the ‘normal’ parameter settings.

### The Features of the Model

#### Activity-dependent suppression of inhibition generates slow seizures

An important question to ask is why is the the inhibitory presynaptic depression factor so important for creating slowly propagation seizures and what modulates this parameter endogenously? The inhibitory presynaptic depression factor determines the rate at which inhibitory synapses adapt. If the factor is higher then the inhibitory synapses will adapt faster allowing the excitatory activity to dominate the inputs to a neuron and for activity to spread across the network. The speed at which the activity propagates across the network is therefore heavily dependent on how much adaptation the inhibitory synapses undergo. Since adaptation is not a fast process compared to the time-scale of a spike the speed of activity propagation ends up being quite low. How the inhibitory presynaptic depression factor is influenced by endogenous network activity is an important question that remains to be answered. In both the low extracellular 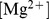 simulation case and the minimal parameter perturbation simulation case we propose that the excess excitation coming from the focus or excitatory neurons drives the inhibitory cells quite vigorously causing the inhibitory presynaptic depression factor to decrease. There are a number of presynaptic and postsynaptic mechanisms [73] that allow synaptic depression to occur. These mechanisms include a reduction in the probability of vesicular release induced by either presynaptic GABA activation [74] or metabotropic glutamate receptor activation [74], [75], depletion of transmitter stores [76] or receptor desensitization [77].

A remaining question is, why doesn’t the excitatory presynaptic depression factor decrease as a result of vigorous activity? In the low extracellular 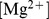 case it may be that pre- and post-synaptic effects of low extracellular 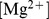 on NMDA receptors prevents this from happening. In the case of ‘normal’ tissue it may be that strong activity drives an increase in extracellular 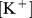 [78] making it easier for excitatory cells to fire even though presynaptic adaptation is more significant. Or, perhaps what happens is that for the right set of network conditions the excitatory presynaptic depression factor does decrease as a result of vigorous activity, but not as much as the inhibitory presynaptic depression factor. This is consistent with the facts that certain cortical inhibitory neurons tend to spike faster than excitatory neurons and that adaptation occurs faster in inhibitory synapses than excitatory synapses under normal conditions [58].

A possible alternative to decreasing the inhibitory presynaptic depression factor to simulate low extracellular 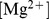 seizures, is to increase the inhibitory reversal potential [66]. Through our simulations it was found that changing the inhibitory reversal potential (i.e. a different fixed value for each simulation) had effects on seizure velocity that were more akin to the GABA antagonist simulations. Rather than exploring the effects of varying a fixed inhibitory reversal potential, it may be possible to more reliably produce slow velocity seizures by simulating activity dependent changes in the inhibitory reversal potential [66], [79].

For the generation of slowly propagating seizures the main point would appear to be the inclusion of slow (relative to spiking) activity-dependent reduction in the efficacy of inhibition [32], [79]. Here we have shown this is the case by modifying inhibitory presynaptic depression, but we believe this would also work using activity dependent changes in the inhibitory reversal potential. This is because the time constants of these dynamics are also slower than those related to spiking, and significant increases in the inhibitory reversal potential and thus a significant weakening of inhibition occur only after multiple spiking wavefronts have passed [66]. Implying that slow, staggered seizure propagation could also be simulated for this case.

#### Network connectivity

A key aspect we explored in this paper is the anatomical connectivity that produces a functional surround suppression. In particular we looked at excitation broader than inhibition and inhibition broader than excitation. In the disinhibition model, we observed that when excitation is broader than inhibition the threshold excitatory-to-excitatory synaptic strength for seizures to spread is much lower than when inhibition is broader than excitation. In the case of the low extracellular 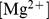 simulations, a slightly greater degree of inhibitory presynaptic depression is required in order to obtain faster seizure spread when excitation is broader than inhibition. This seems contrary to the disinhibition result, however, this may depend on the operating point of the model when excitation is broader than inhibition, as the excitatory-to-inhibitory and inhibitory-to-excitatory weights need to be higher in order to create functional surround suppression. Generally, it was found that either form of anatomical connectivity could be used to produce the full range of empirically observed propagation velocities. Although some anatomical evidence points towards excitation being broader than inhibition, we believe that this still critically depends on the laminar layer(s) of cortex to be modelled, and that there may be some scenarios where inhibition could be broader than excitation depending on the spatial scale of interest.

#### Spatial correlations and disordered waves

The analysis of spatial coherence via tracking the MCC revealed that decreases in MCC corresponded to increases in the rate of wavefront occurrence in the GABA antagonist simulations with zero inhibition. In the case of the GABA antagonist simulations with non-zero inhibition, decreases in the MCC also coincided with increases in disorder. For the low extracellular 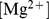 simulations the waves were usually disordered and there where no obvious changes in the MCC apart from the change observed as the seizure front expands. This MCC analysis was inspired by a study of spiral waves (velocity of up to 60 mm/s) in an anaesthetised *in vivo* animal model of sleep-like states [67]. They observed that MCC decreased when spiral waves occurred. Although we have not focused on spiral waves here, spiral waves do correspond to a more disordered state than regular periodic waves and therefore our findings are consistent with theirs.

Given that all membrane potentials were initially set to the resting potential for each simulation, there are two ways we expected disordered activity patterns to occur in our simulations. The first is through fixed (i.e. time independent) perturbation of both the Gaussian excitatory and inhibitory connection strength kernels in Equation 7. The second is that the inhibitory neurons are positioned at the position of every second excitatory neuron along both the x and y axes. Given that the focal input in our simulations is positioned at the central 4×4 position in the 50×50 excitatory neuron grid, this means that the inhibitory neurons are positioned asymmetrically with respect to the group of excitatory neurons that receive this input. If a 3×3 or 5×5 input grid was used there would be no asymmetry, but then the input would not be positioned directly at the centre of the 50×50 excitatory neuron network. It might have been better to position each inhibitory neuron at the centre of a 2×2 array of excitatory cells. This would have given symmetry for a 4×4 central input. If this was done disorder could have been controlled for more, through the degree of perturbation of the connection strengths, however, the main purpose of this paper is to look at how velocity changes in networks where disordered wave patterns can occur, rather than to better understand the causes of order and disorder in the wave patterns and their resultant effects on seizure propagation velocity.

The presence of disordered waves in our network appears to depend on the degree of inhibition. For the GABA antagonist case, the stronger the inhibition, the greater the disorder and the slower the wave propagation. For the low extracellular 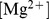 results, inhibition is always present but gets modulated by the depression and as such disordered waves generally occurred for all low extracellular 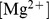 results. Thus in our model, disordered waves were linked to slower seizure propagation velocities, but our simulation analysis does not try to disentangle the effects of disorder (due to the network connection strength perturbations and inhibitory/excitatory cell alignment) and the effects of changes in inhibition on propagation velocities. In order to disentangle these two effects one could avoid disorder in the network activity by shifting the positioning of our inhibitory neurons relative to excitatory neurons to create symmetry and by not perturbing the Gaussian connectivity strengths. However, we decided not to analyse this as we expect the main effects on seizure propagation velocity to come from changes in inhibition and synaptic depression, in part because the degree of disorder was dependent on the degree of inhibition. Moreover, the more interesting and biologically realistic scenario involves disordered patterns and perturbed connection strengths, without which patterns such as spiral waves would not occur [12].

#### Simulation caveats and degrees of realism

The next point to make is that the computational model presented has a large number of parameters, with a change in any having an impact on seizure propagation speeds. It is indeed restrictive to produce data for such a large multi-dimensional parameter space which is why the dimensionality of the parameter space was reduced with a focus being placed on synaptic connectivity, synaptic strength and presynaptic depression. These parameters were focused on because through simulation they showed the greatest influence on seizure propagation speeds and many of the other parameters have been determined empirically, extensively throughout the literature and were taken as constants.

In our model, we simulated seizure activity in a simplistic manner by applying an external current, inserted into a central 4×4 cluster of excitatory neurons for network activity to be sustained. This method is utilised in several neural network simulation studies [12], [80], [81]. Moreover, we considered ‘normal’ tissue to be that which does not allow waves to propagate beyond the central 4×4 cluster of neurons. Thus the model does not generate spontaneous waves of activity seen in the ‘normal’ brain, or at least for the purposes of this study we do not consider such waves to be ‘normal’. Examples of spontaneous activity include persistent ongoing complex behaviour [82], [83] with neural activity being self-sustaining when the brain enters a mode where it is ‘disconnected’ from external stimuli [84]. Moreover, travelling waves have been observed in normal cortical tissue [85]–[87]. Rubino suggests that these waves mediate information transfer in the motor cortex [88] and Ermentrout claims that they may have a computational role [89].

Our simplistic approach is a reasonable first step for two reasons. First it simplifies the model and allows us to focus directly on controlling the spread of activity. Second, this approach is reasonable if we consider layer 4 of primary visual cortex where spatial resolution of the input image needs to be preserved and activity should not spread beyond where the inputs are present. Only through subsequent hierarchical processing can integration of information across space take place in a context dependent manner. A potential extension of our model is to enable it to generate spontaneous seizure-like activity. We have avoided this here because spontaneous seizures could emerge anywhere in the network, instead of from the central region, making it harder to study the speed of seizure propagation in the model. The model produced by Stratton was successful in generating complex, non-periodic network activity with the the removal of external input where the power spectrum of the simulated EEG/LFP is similar to that observed in recordings from human cortex [90]. The models of Rothkegel and Lehnertz [13], [91] were also capable of producing spontaneous seizure activity. Physiologically, the combination of bursting and excitatory recurrence [92]–[94] appear important for the spontaneous initiation of seizure-like events and will need to be taken into consideration when simulating spontaneous emergence of seizures.

An additional simplification in our models is the use of integrate and fire neurons as opposed to compartmental neurons. While compartmental models are good at telling us which parts of cells could be involved in starting or propagating seizures, it is difficult to delineate with networks of compartmental neurons whether it is the cell properties or network properties that are the most critical. By simplifying cells to the IAF case, one can familiarize oneself more with critical network properties before adding in greater cellular detail.

### Comparison to other Models

We explored the speed of propagation of seizure-like activity in a 2-D network containing both excitatory and inhibitory populations, considering both the low extracellular [Mg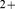] and disinhibition models of seizure spread in the same computational model. With respect to our results, Compte et al. [38] provided the closest study to ours in that they explore the velocities of slow waves linked to ‘up’ and ‘down’ states and fast waves linked to the disinhibition model in a 1-dimensional spatial model of a layer 5 prefrontal ferret cortex which includes excitatory and inhibitory populations. In their model ‘up’ states emerged by recurrent excitation and the ‘down’ states resulted from slow activity-dependent K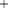 currents. This was done to model the results seen in *in vitro* slice models of slow oscillations [95]. With regard to seizures, however, Trevelyan et al. [22], [23] have shown that the failure of inhibition appears to produce both slow (0.1 mm/s) and fast (10–15 mm/s) waves of epileptic activity in low extracellular [Mg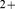] slices, depending on the degree of inhibitory failure. Our simulations are consistent with Trevelyan et al. 's findings, in our computational model slow seizures (0.1 mm/s) are linked to small increases in depression of synaptic inhibition and faster seizures (10–20 mm/s) are linked to further removal of inhibition via significant increase in the degree of synaptic depression. Many other computational modeling papers on either disinhibition or slow waves [37], [96]–[101] explore the propagation of seizure-like waves in networks with primarily 1-dimensional spatial structure (or no spatial structure at all) and often focus less on how parameter changes effect velocity of propagation. In our results section and above we have discussed the influence inhibition has on the degree of disorder in 2D wave patterns. We have also explored the influences of a functional Mexican hat centre-surround with surround suppression that can emerge from two forms of anatomical connectivity depending on the relevant synaptic weight values: inhibition broader than excitation, and excitation broader than inhibition.

#### A novel low extracellular [Mg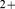] simulation

With respect to the low extracellular [Mg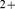] model [22], [23], [93] we explored parameter changes that produce the observed slow velocity seizure-like activity. Trevelyan et al. [22] showed that low extracellular [Mg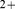] seizures involve slow modular step-wise propagation. Pairwise recordings show recruitment and failure of inhibition are coincident. Cells experience both excitation and feedforward inhibition until inhibition fails. They did not address why inhibition fails, however, recently it has been demonstrated that specific fast spiking inhibitory neurons are involved in containing the spread of excitation in a seizure [102]. In another paper, Trevelyan et al. [23] showed that strength of feedforward inhibition (number of preictal inhibitory barrages) correlates with velocity of propagation. In both of the Trevelyan et al. papers they only speculate on the mechanisms which cause inhibition to fail. In our paper we explore through a computational model the mechanisms through which inhibition can fail, especially to produce slowly propagating seizure-like waves. We found that parameters linked to synaptic depression of inhibition were critical in producing slow waves. To the best of our knowledge, our model is the first to simulate Jacksonian-March type propagation observed in 2-dimensional low extracellular [Mg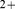] slices. Compte et al. [38] provided similar simulations in 1D but in the different context of ‘slow wave’ slices as mentioned above. It is expected that in the Compte et al. model the slow wave propagation velocity is more to do with transitions between ‘up’ and ‘down’ states. Whereas in our model of low extracellular [Mg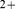] slices, excitatory wavefronts would travel faster but keep coming up against a wall of inhibition until that wall fails via increased inhibitory synaptic depression and activity can propagate further out. This leads to slow measured average propagation velocities across the full extent of the network.

#### GABA antagonist simulations extend knowledge in 2D space

With respect to the disinhibition model we explored the parameter changes that produce the observed fast velocity seizure-like activity. Various studies have explored disinhibition *in vitro* [26], [35], [37], [94], [103], [104]. Pinto et al. [26] showed that propagation velocity increased with increase in injected input current used to initiate discharges. In our simulations we found that for currents above 3000 pA the velocity was independent of current. For the range 800–3000 pA the velocity decreased as current increased. Anything below 800 pA resulted in no propagation. Chagnac-Amitai and Connors [103] found that for low amounts of GABA blockers complex waves form, whereas for high concentrations the waves are regular without decrement or reflection. As mentioned above, in our simulations we also found that order of the waves increased as the inhibitory weight was slowly decreased.

There have also been several computational models of disinhibition [12], [35]–[38]. Miles et al. [35] and Traub and Miles [36] showed for very similar 2D models of a slice of area CA3 of hippocampus, that decreasing inhibition or increasing the spatial extent of excitation during disinhibition lead to increases in propagation velocity. They also demonstrated that the velocity of propagation and the extent of surround inhibition seen in vitro could be accounted for by the model. They did not give much consideration of spatial properties of the wave such as the analysis of the relationship between inhibition and pattern order that we have discussed in the results. We have also explored the influence of the strength of excitatory and inhibitory synapses and the spatial extent of excitation in more detail, and illustrated for a large range of parameters that there is an effective threshold velocity linked to the balance of excitation and inhibition beyond which seizures can spread. Moreover, this effective threshold exists even when no inhibition is present indicating that other features such as the integration of input spikes are also important. Traub and Miles [36] also focused on excitation broader than inhibition, whereas we have compared two connectivity scenarios: excitation broader than inhibition and inhibition broader than excitation.

As mentioned above, Compte et al. [38] also explored 1D spatial simulations of the disinhibition model. They found that either an increase of the inhibitory conductance onto excitatory cells or of the excitatory conductances onto inhibitory cells gradually decreases the wave propagation speed. They also reported that increasing the leak conductance increases the velocity of propagation. We have explored these parameters to some extent, in particular our changes in synaptic strength are similar to their changes in conductance and our 2D results are consistent with their 1D results.

Ursino and La Cara [12] looked at seizure propagation patterns in a 2D network of neurons but each neuron projected both excitatory and inhibitory synapses thus violating Dale’s law. We extended their results by including both excitatory and inhibitory neurons and synaptic depression mechanisms, and putting more focus into analyzing the velocity of propagation. Also Ursino and La Cara only focused on the disinhibtion model and did not explicitly explore the low extracellular [Mg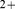] model. Nevertheless, their work provides an interesting exploration of the spatiotemporal characteristics of seizure-like dynamics produced by their model. In particular, they demonstrated that increases in the strength and spatial extent of excitatory versus inhibitory synapses lead to propagation of seizure-like activity that produced different forms of LFP (i.e. irregular large amplitude rhythms, quasi-sinusoidal rhythms, low amplitude high-frequency discharges) and wave patterns (i.e. periodic/ordered, disordered and spiral) for different parameter sets. In our study we have focused more on propagation velocity, but many features of our simulations were consistent with Ursino and La Cara. Such as the existence of periodic/ordered, disordered and spiral wave patterns depending on the parameters used.

Other network models mainly focus on mechanisms of seizure initiation or seizure spread through networks, layers or regions [13], [78], [105]–[112], but not 2D sheets of neurons topologically organised with a functional Mexican-hat connectivity structure. Models that have considered 2D sheets of neurons in more detail are of the mesoscale/mean-field nature [9], [10]. These mesoscale studies by Kramer and colleagues focus on accounting for the frequency of maximum power and the speed of spatial propagation of voltage peaks estimated from human intracranial EEG. At the meso/macro-scale the speed of spatial propagation of voltage peaks was 2 m/s. Kramer and colleagues propose that this rapid propagation arises from white matter connections between regions, whereas the slower propagation speeds seen in animal slices (0.1–100 mm/s) results from local connections within the cortical grey-matter. In their model, seizures emerge through affecting the balance of excitation and inhibition, somewhat akin to our simulations of the GABA antagonist slice model. Our simulations in 2D centre-surround recurrent networks are consistent with the framework of Kramer et al. and the modifications of synaptic depression shown here, also provide a way for exploring slow velocity seizure propagation in mesoscopic models of human data.

## Supporting Information

Video S1
**GABA antagonist case with zero inhibition and 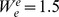 and corresponding to** **Figures 1A** **and** **2A** **.**
(MP4)

Video S2
**GABA antagonist case with zero inhibition and 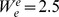 and corresponding to** **Figures 1B** **and** **2A** **.**
(MP4)

Video S3
**GABA antagonist case with zero inhibition and 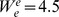 and corresponding to** **Figures 1C** **and** **2A** **.**
(MP4)

Video S4
**GABA antagonist case with non-zero inhibition, 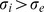 and 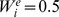 and corresponding to** **Figures 3A** **and** **2B** **.**
(MP4)

Video S5
**GABA antagonist case with non-zero inhibition, 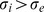 and 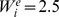 and corresponding to** **Figures 3B** **and** **2B** **.**
(MP4)

Video S6
**GABA antagonist case with non-zero inhibition, 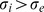 and 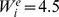 and corresponding to** **Figures 3C** **and** **2B** **.**
(MP4)

Video S7
**GABA antagonist case with non-zero inhibition, 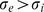 and 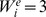 and corresponding to** **Figures 4A** **and** **2C** **.**
(MP4)

Video S8
**GABA antagonist case with non-zero inhibition, 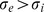 and 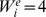 and corresponding to** **Figures 4B** **and** **2C** **.**
(MP4)

Video S9
**GABA antagonist case with non-zero inhibition, 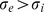 and 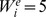 and corresponding to** **Figures 4C** **and** **2C** **.**
(MP4)

Video S10
**Low extracellular [Mg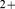] case with 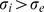 and corresponding to** **Figures 7** **and** **2D** **.**
(MP4)

Video S11
**Low extracellular [Mg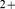] case with 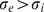 and corresponding to** **Figures 8** **and** **2D** **.**
(MP4)
